# Activity of *Bdellovibrio Hit* Locus Proteins, Bd0108 and Bd0109, Links Type IVa Pilus Extrusion/Retraction Status to Prey-Independent Growth Signalling

**DOI:** 10.1371/journal.pone.0079759

**Published:** 2013-11-05

**Authors:** Michael J. Capeness, Carey Lambert, Andrew L. Lovering, Rob Till, Kaoru Uchida, Roy Chaudhuri, Luke J. Alderwick, David J. Lee, David Swarbreck, Susan Liddell, Shin-Ichi Aizawa, Renee Elizabeth Sockett

**Affiliations:** 1 School of Life Sciences, University of Nottingham, Nottingham, United Kingdom; 2 School of Biosciences, University of Birmingham, Birmingham, United Kingdom; 3 Department of Life Sciences, Prefectural University of Hiroshima, Shobara, Japan; 4 Institute of Integrative Biology, University of Liverpool, Liverpool, United Kingdom; 5 The Genome Analysis Centre, Norwich, United Kingdom; 6 Division of Animal Sciences Proteomics Laboratory, University of Nottingham, Nottingham, United Kingdom; University of Strathclyde, United Kingdom

## Abstract

*Bdellovibrio bacteriovorus* are facultatively predatory bacteria that grow within gram-negative prey, using pili to invade their periplasmic niche. They also grow prey-independently on organic nutrients after undergoing a reversible switch. The nature of the growth switching mechanism has been elusive, but several independent reports suggested mutations in the *hit* (host-interaction) locus on the *Bdellovibrio* genome were associated with the transition to prey-independent growth. Pili are essential for prey entry by *Bdellovibrio* and sequence analysis of the *hit* locus predicted that it was part of a cluster of Type IVb pilus-associated genes, containing *bd0108* and *bd0109*. In this study we have deleted the whole *bd0108* gene, which is unique to *Bdellovibrio*, and compared its phenotype to strains containing spontaneous mutations in *bd0108* and the common natural 42 bp deletion variant of *bd0108*. We find that deletion of the whole *bd0108* gene greatly reduced the extrusion of pili, whereas the 42 bp deletion caused greater pilus extrusion than wild-type. The pili isolated from these strains were comprised of the Type IVa pilin protein; PilA. Attempts to similarly delete gene *bd0109*, which like *bd0108* encodes a periplasmic/secreted protein, were not successful, suggesting that it is likely to be essential for *Bdellovibrio* viability in any growth mode. Bd0109 has a sugar binding YD- repeat motif and an N-terminus with a putative pilin-like fold and was found to interact directly with Bd0108. These results lead us to propose that the Bd0109/Bd0108 interaction regulates pilus production in *Bdellovibrio* (possibly by interaction with the pilus fibre at the cell wall), and that the presence (and possibly retraction state) of the pilus feeds back to alter the growth state of the *Bdellovibrio* cell. We further identify a novel small RNA encoded by the *hit* locus, the transcription of which is altered in different *bd0108* mutation backgrounds.

## Introduction

*Bdellovibrio bacteriovorus* is a Gram-negative bacterium which preys on other Gram-negative bacteria by attaching to the prey cell in an invasive process that requires Type IVa pili, including the main PilA fibre protein encoded by gene *bd1290* [1,2]. After *Bdellovibrio* enters the periplasm, the prey cell is rounded up to form a bdelloplast structure, wherein the *Bdellovibrio* releases a cocktail of enzymes to hierarchically break down the components of the prey cytoplasm and inner membrane [3-5], which are utilized by the invaded *Bdellovibrio* to grow and septate. The *Bdellovibrio* then break out of the bdelloplast by controlled lysis of the remaining outer membrane and wall and do not replicate until they invade prey and repeat the predatory cycle. 

*B. bacteriovorus* retains the genes to allow growth prey-independently (so called HI for Host-Independent cells; versus cells growing as predators which are called HD for Host-dependent). This HI growth occurs in the absence of prey cells and on protein-rich media. Curiously only a low percentage of HD predatory cells switch naturally to HI growth when plated on rich media. This phenomenon was first observed by Stolp and Starr [6] for HI colonies growing saprophytically on lawns of *E. coli* at low frequencies. They were first isolated by Shilo and Bruff [7] and shown to be derived from host dependent (HD) *Bdellovibrio*, it being noted that a large number of HD cells, 10^6^ - 10^7^, were required for a few colonies to form. Work by Cotter and Thomashow [8] attributed the HI phenotype, and the amount of cells required to generate these HI isolates, to a mutational event in the *hit* (host-interaction) locus in the *Bdellovibrio* genome, and noted that complementing with the same locus restored plaquing efficiency, which was seen to be lowered but not abolished in HI *Bdellovibrio*. Cotter and Thomashow also first reported a *hit* mutant with a deletion of 42 bp in gene *bd0108*, flanked at each end by 10 bp direct repeats. They also noted that in some HI isolates, there were no mutations in the *hit* locus, hypothesizing that there must be a second ‘HI’ generating mutation elsewhere in the genome [8].

The majority of all the HI-growth-associated mutations in the *hit* locus are within the equivalent to *bd0108* in the genome-sequenced strain of *B. bacteriovorus* HD100. Wurtzel and co-workers [9] showed that 89% of HI *Bdellovibrio* isolates were mutated in *bd0108*, with the other 11% of mutations presumably residing elsewhere in the genome. A common mutation observed was the deletion of 42 bp from the middle of *bd0108* (46% of the mutations they reported in *bd0108* were this deletion); the mutation also previously isolated by Cotter and Thomashow [8]. Not only does their study suggest that the 42 bp *bd0108* deletion is of importance as it occurs readily, but also that mutation in *bd0108* can be sufficient for HD conversion to HI, though is not absolutely required [9].

Gene *bd0108* was found to lie in the genome next to genes predicted to have a function in Type IVb flp pilus assembly [10]. Further work by Schwudke and co-workers [11] hypothesized that *bd0108* is involved in Type IVb (flp) pilus formation; though no Bd0108 protein, or indeed their proposed flp pilins: proteins Bd0118/0119, were found in *Bdellovibrio* cell envelope fractions that were analysed by SDS-PAGE and LC-MS, which did detect the Type IVa pilin PilA (Bd1290). 

The only targeted mutagenesis to date of *bd0108* has been made by transposon insertion [12], which showed that the gene product has a role in HI growth and its mutation results in *Bdellovibrio* that produce faint cloudy plaques on prey lawns; while complementation with the wild-type gene restored the wild-type, clear plaquing phenotype. Although this was a targeted mutation, the transposon inserted after 193bp (64 codons) of the 303bp gene not necessarily fully inactivating the function of Bd0108. Markerless deletion, although attempted by Roschanski and co-workers, proved elusive.

Despite the repeated natural isolation of HI strains carrying a mutation in *bd0108*, no mechanistic basis for its role in host-independent growth-switching has been discovered. To this end, for both *bd0108* and its neighbour *bd0109*, we initiated markerless deletion studies, complementation tests, fluorescent tagging of products for cellular address-determination, transcriptional analyses and interaction studies of the proteins. From this data we propose a model by which Bd0108 and Bd0109 interact to govern pilus assembly and we suggest that this is coupled to the growth and the development processes of the *Bdellovibio* cell, possibly via small RNA signalling.

## Results

### A markerless deletion mutant of *bd0108* forms HI colonies and completes predation when offered prey but a *bd0109* deletion is lethal

Markerless deletion of all but 6 codons (coding MGKRQ-) of the *Bdellovibrio bd0108* gene from the wild-type strain HD100 was achieved by delivery of the suicide plasmid into predatory HD *Bdellovibrio* and immediate plating of large numbers of cells onto PY media to allow HI growth. Despite multiple rigorous attempts, deletion mutants were only ever recovered from HI grown exconjugants on PY media and not from predatorily HD-grown exconjugants on prey-lawn overlay plates. This was despite screening exconjugant numbers far in excess of those successfully yielding HI-derived mutants (Table **1**) and far in excess of those successfully yielding mutants in other genes [13,14]. The reason that only exconjugants which had reverted to wild-type (rather than ∆*bd0108*mutants) were recovered in HD-grown cultures may be because wild-types out-competed any ∆*bd0108* mutants which were less efficient at predation (see below). Several rounds of sucrose selection, over more than a week, are necessary to recover exconjugants. 

**Table 1:** The numbers of exconjugant *B. bacteriovorus* HD100 which yielded a double crossover (giving either wild-type revertant or deletion mutant); the outcomes suggest that it is only possible to obtain a deletion mutant of *bd0108* by recovering HI *Bdellovibrio* and that *bd0109* may be essential for *Bdellovibrio* viability in the HD and HI growth conditions that we used.

	Δ*bd0108*		Δ*bd0109*	
Number of deletion strains	HD = 0	HI = 2	HD = 0	HI = 0
Number of revertants	HD =343	HI = 46	HD =207	HI = 217
Number of Conjugations	4		8	

**Figure 1:**
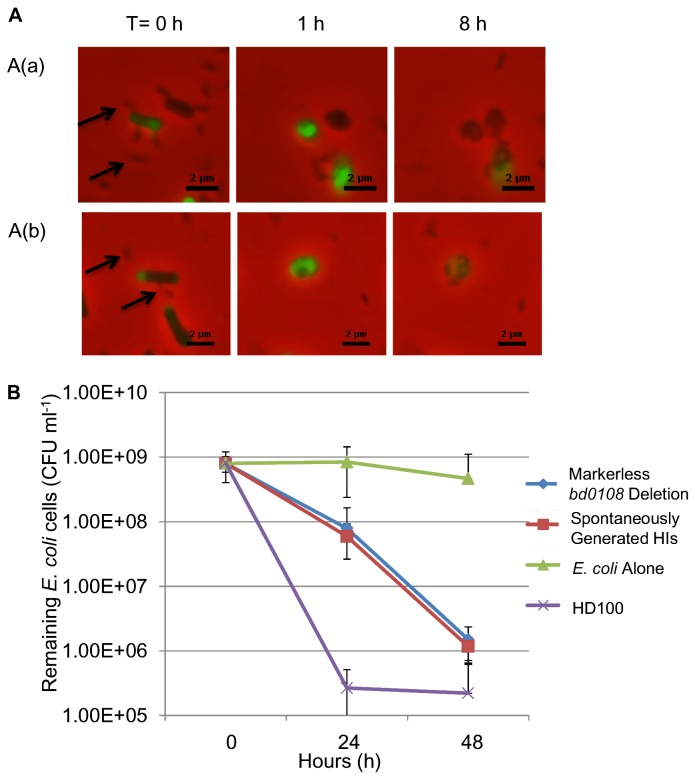
Predation of HI strains compared to predatory HD100. Time-lapse microscopy of individual wild-type HD100 cells (**A**(**a**)) and cells of the markerless deletion mutant of *bd0108* (A(b)) preying upon the *E. coli* strain pMAL-p2_mCherry with a fluorescent periplasm. At T=0 there is attachment of free swimming *Bdellovibrio* in both strains, with invasion occurring within 2 frames (5 minutes) for most cells observed. Arrows indicate both free swimming and attached *Bdellovibrio* cells. By 1 hour the *Bdellovibrio* is established in the periplasm and begun to grow as a filament. By 8 hours the growing filaments of both the wild-type and deletion strains have septated to form single progeny and the prey bursts releasing them. Predation was carried out on a 1% Agarose pad with images acquired every 2.5 minutes. **B**. Reduction of *E. coli* numbers in a predatory lysate comparing wild-type HD100 or spontaneously generated HIs with the markerless deletion of *bd0108* strains. The spontaneously generated HI strains included some with a variety of point mutations or the common 42bp deletion in *bd0108* and some with a wild-type copy of *bd0108*. HI cultures readily prey upon *E. coli* in liquid cultures, reducing prey numbers by several logs in 2 days with predation by the population as a whole appearing to be slightly slower than wild-type HD100. All HI strains were grown axenically, independently of prey cells before the experiment.

 Interestingly, deletion of the adjacent gene, *bd0109*, was not possible under HI or HD growth conditions; this is discussed later in the results section 2 ∆*bd0108* isolates were obtained (∆*bd0108#1 and ∆bd0108#2*) and confirmed to contain the expected deletion of *bd0108* by PCR and subsequent sequencing of the PCR product, Southern blot analysis and RT-PCR expression analysis of *bd0108* (data not shown). Both isolates grew Host Independently (HI) on PY media both on agar and in liquid culture without any specific morphological abnormalities compared to other HI isolates. When offered *E. coli* prey, the mutants were observed entering, growing within, and lysing prey cells when viewed by time lapse microscopy on a 1% agarose pad (Figure **1A**). They completed this predatory cycle within 7-10 hours which is comparable to the wild-type strain HD100 in these conditions (the usual predatory cycle in liquid culture lasts 2-5 hours, but is longer observed in these time-lapse experiments with the cells immobilized on agarose). Invasion time (from first appearing to move into the prey cell, until fully inside) appeared to be within 2 frames (each frame 2.5 minutes, so less than 5 minutes invasion time) for ∆*bd0108* cells observed (n=21), which is similar to the 4.4 minutes ±1.18 recorded for wild-type HD100 [13].

The ∆*bd0108* strains along with natural, spontaneous HI isolates were used in this study; HID2, HID26 (both with no *bd0108* mutation) HID6, HID13, HID18 (with point mutations in *bd0108* at 217, 3 and 211 bp corresponding to amino acids 72, 1, and 70, respectively, Figure **S1**) and HID22 (*bd0108∆*42bp). All grew on plates and in liquid media as HI strains but could also carry out predation in liquid culture. *Bdellovibrio* cell numbers were matched by total protein (due to diverse cell morphologies [15]), using the Lowry assay which allows an “instant” enumeration, which we have used in previous work [15-17]. This is required for *Bdellovibrio* which are too small (HD cells); or too diverse in cell sizes (HI cells); to produce a meaningful optical density, The ∆*bd0108* strains preyed as rapidly upon *E. coli* as these other HI strains as measured by reduction in *E. coli* CFU numbers (Figure **1B**). All HI strains were less efficient at predation than the wild-type predatory HD100 strain. This lowered efficiency may be due to a proportion of the population growing as HI cells rather than predatorily. The HI strains reduced the *E. coli* numbers (although more slowly than the predatory HD100) by ~3 log_10_ within 48 hours which is comparable to ~3.5 log_10_ reduction by HD100 demonstrating that HI strains can still carry out predation significantly. Our observation that all HI strains retained predatory capacity is in agreement with Medina and co-workers [18] and with previous reports [19,20] that HI strains were predatory in liquid culture but often not capable of forming plaques. 

### Predation by both the ∆*bd0108* and spontaneous *bd0108* mutants is enhanced by complementation with wild-type *bd0108* and prey-independent growth becomes inhibited

**Figure 2:**
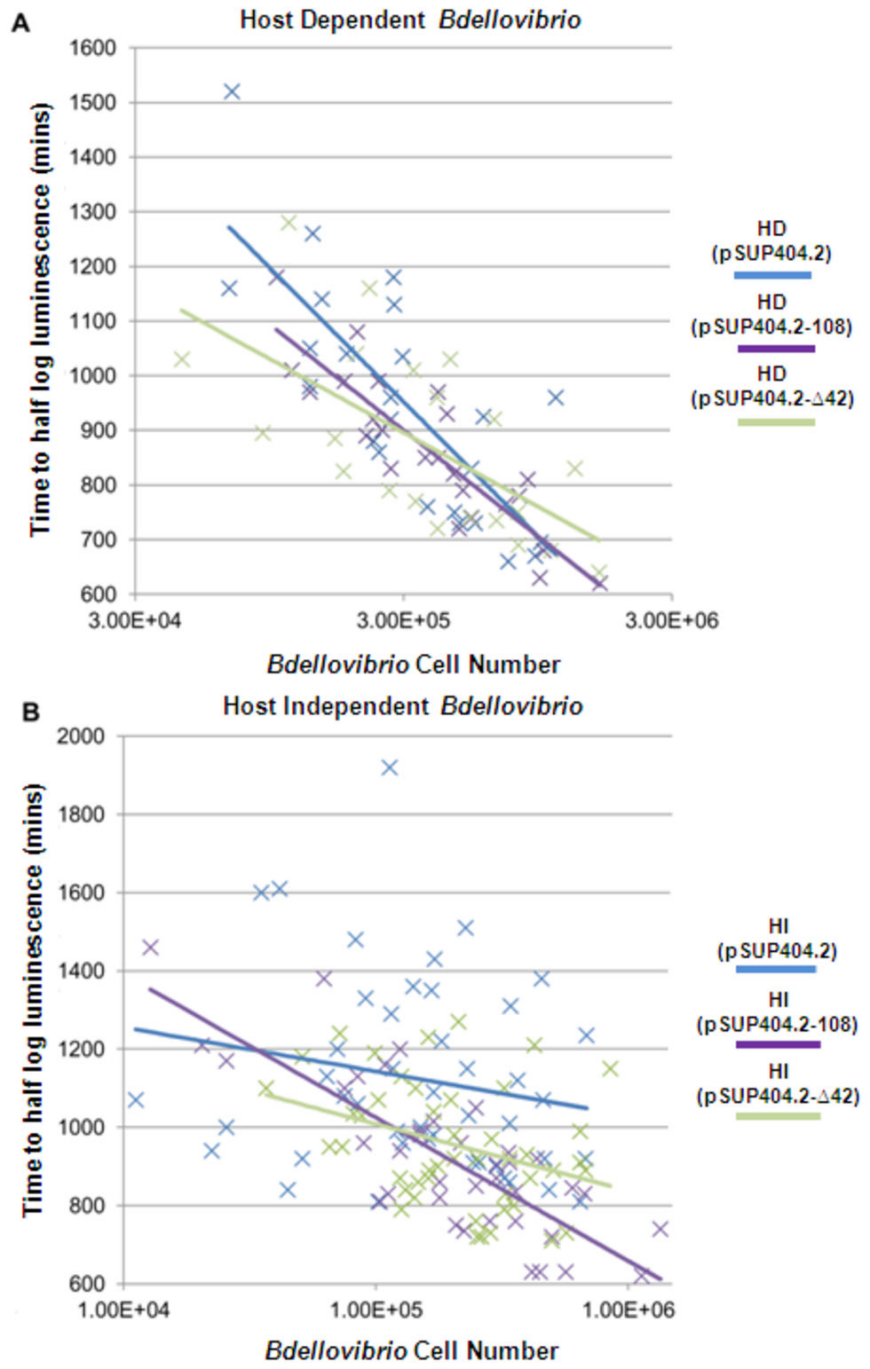
Luminescent prey assay of predation efficiency for Host Dependent and Host Independent strains. For Host Dependent cells (**A**) with a genomic copy of the wild-type *bd0108* gene, carrying the pSUP404.2 plasmid encoding either the wild-type *bd0108* gene or the 42 bp deletion variant of *bd0108* had no effect on predation compared to those carrying the empty pSUP404.2 plasmid. This assay shows the typical result of logarithmically faster reduction in luminescence with more *Bdellovibrio* initially added and shows that the extra copies of *bd0108* or the 42 bp deletion variant have no significant effect on this. For Host Independent cells (**B**) there is not the same proportional decrease in luminescence when more cells are added supporting the hypothesis that a proportion are growing axenically as HIs rather than predatorily. HI mutants carrying the pSUP404.2 plasmid encoding the wild-type *bd0108* gene, however, restore the typical initial-cell-number to drop in luminescence relationship seen in the HD wild-type. This suggests that the presence of a wild-type *bd0108*, but not the 42 bp deletion variant, *in* *trans* restores the HI cells to a predatory lifestyle.

HI grown *Bdellovibrio* are morphologically diverse and this makes matching their cell numbers difficult as discussed in the previous section. To get around this we used a predation assay on standardised numbers of luminescent *E. coli* prey over the course of 48 hours. From this it was possible to assay the efficiency of predation as it correlated with the initial numbers of starting *Bdellovibrio* (Figure **2**), without the need for matching initial amounts by centrifuge concentration such as Percoll gradients [21] which could have a profound effect on the physiological state of the cells. This method has previously been used to measure more subtle deficiencies in predation of populations of mutant *Bdellovibrio* [22-24] and also avoids any potential bias in matching cells of different size by total protein. 

We used this method to determine the extent to which the spontaneous HI isolates and the markerless *bd0108* deletion mutant could complete predation when complemented with a replicating plasmid pSUP404.2 [25] containing variant *bd0108* genes cloned in with 200 bp of flanking DNA. Using pSUP404.2 (empty vector), pSUP404.2-108 (wild-type *bd0108*) and pSUP404.2-Δ42 (the common 42 bp deletion variant of *bd0108*) it was also possible to ascertain the effect encoded by extra copies of these two different forms of *bd0108* genes in the wild-type HD *Bdellovibrio bacteriovorus* strain. The pSUP404.2 vector was reported by Roschanski and co-workers to have a copy number of seven per cell in HD *Bdellovibrio* cells [25].

Predation by *Bdellovibrio bacteriovorus* HD100 wild-type, carrying any of the *bd0108* variants on the pSUP404.2 plasmid was not enhanced or reduced in the efficiency compared to cells carrying an empty plasmid alone (Figure **2A**). This assay shows the typical result of logarithmically faster reduction in luminescence with more *Bdellovibrio* initially added and shows that the extra copies of variant or wild-type *bd0108* have no significant effect on this when wild-type *bd0108* is already expressed. 

For the HI strains, both spontaneous and Δ*bd0108* deletion strains, there was an interesting deviation from this typical predation plot shape, namely that with more added initial *Bdellovibrio*, there was not the expected increase in rate of prey luminescence reduction which relates to predation of the luminescent prey cells (Figure **2B**). This is likely a result of an increased amount of *Bdellovibrio* in the HI growth mode, using released prey debris. For HI growth, cell density dependence (akin to a quorum sensing phenotype) has been reported previously [7] and we have previously noticed a lack of successful growth of HI cultures inoculated at low cell density. HI mutants carrying pSUP404.2-108 however were complemented in terms of predation efficiency. This complementation restored the typical steeper gradient to the line showing initial-*Bdellovibrio*-cell-number to drop in prey luminescence relationship to that seen for the HD100 wild-type (Figure **2**). This supports the hypothesis that the presence of the wild-type Bd0108 is increasing predation by reducing the percentage of the population that is in the HI growth state.

**Figure 3:**
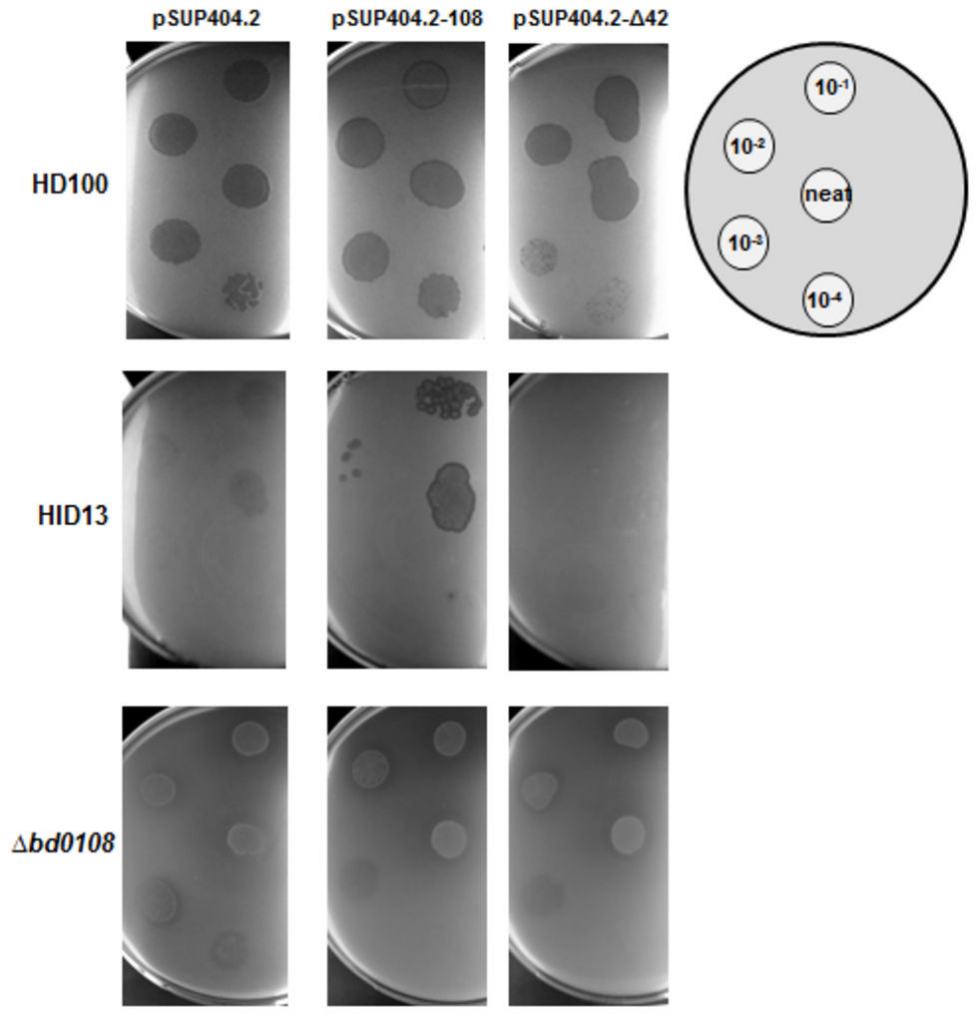
Plaque assay of predation by Host Independent mutants compared to HD100. Dilution of predatory cultures of *Bdellovibrio* strains on double layer YPSC overlay plates containing prey *E. coli* S17-1 in the top layer. Dilution was carried out in Ca/HEPES with 10 µl of the resulting dilution series spotted at positions indicated in the diagram. Plates show there is no significant effect on HD100 having extra copies of the *bd0108* gene or the 42 bp deletion variant of *bd0108* on plaquing ability. Complementation tests for the HID13 strain (ATG->ATA mutation in *bd0108*) with the plasmid pSUP404.2 containing the wild-type *bd0108* gene showed an increase in plaquing, however this was not observed for the Δ*bd0108* deletion strain. Plates are representative of at least three independent repeats.

To compare our data to that of Roschanski and Strauch [12] for a *bd0108* transposon mutant which they had complemented with a wild-type *bd0108* gene, plaquing capability of each strain was also measured. To do this, we used serial dilutions to 10^-4^ of complemented strains grown in predatory cultures and spotted 10 µl upon dual layer overlay plates containing *E. coli* in the top layer of agar. For the spontaneous HI isolates tested, HID13 (and HID22 - data not shown) complementation with pSUP404.2-108 enhanced the plaquing capability compared to both that of pSUP404.2 alone and pSUP404.2-Δ42 (Figure **3**). It had previously been reported that complementation of the reduced plaquing in HI mutants could be achieved by crossover [26] as well as *in trans* by the addition of wild-type *bd0108* [12]. We observed a similar complementation effect, except we found that plaquing ability was a very variable phenotype. Here we show a strain with a *bd0108* point mutation was clearly complemented (as in previous reports), but the Δ*bd0108* markerless deletion strains had strong HI growth, masking the area of clearing in both control and complemented strains and thus making the assay hard to read. It was also noted that in some cases, the lower dilution spots of *Bdellovibrio* gave clearer plaquing, again indicating that the presence of more HI cells in the higher dilutions was impeding predatory growth. 

We have previously noted variable cloudy/clear plaque appearance from spontaneous (not directed) HI mutants of *Bdellovibrio*. We isolated eleven spontaneous HI strains, derived from HD100, in previous studies. These were tested for plaquing ability (without plasmids present) by spotting onto dual layer overlay plates containing *E. coli* in the top layer of agar. Three of these gave clear plaques, with the other eight strains forming more cloudy plaques similar to those seen for the uncomplemented control of HID13 (with pSUP404.2 only) in Figure **3**. These observations are similar to those of other investigators who also noted the variable abilities of HI strains to form plaques [19,20].The fact that the number of HI cells altered the plaquing efficiency in our *Δbd0108* complementation experiments, but that a threshold of cells is needed before plaques are seen, is informative to spontaneous HI phenotypes. It suggests that differences in HI strain growth rate, or cell-cell interaction differences, can result in differences in plaque morphology in spontaneous HI strains.

**Figure 4:**
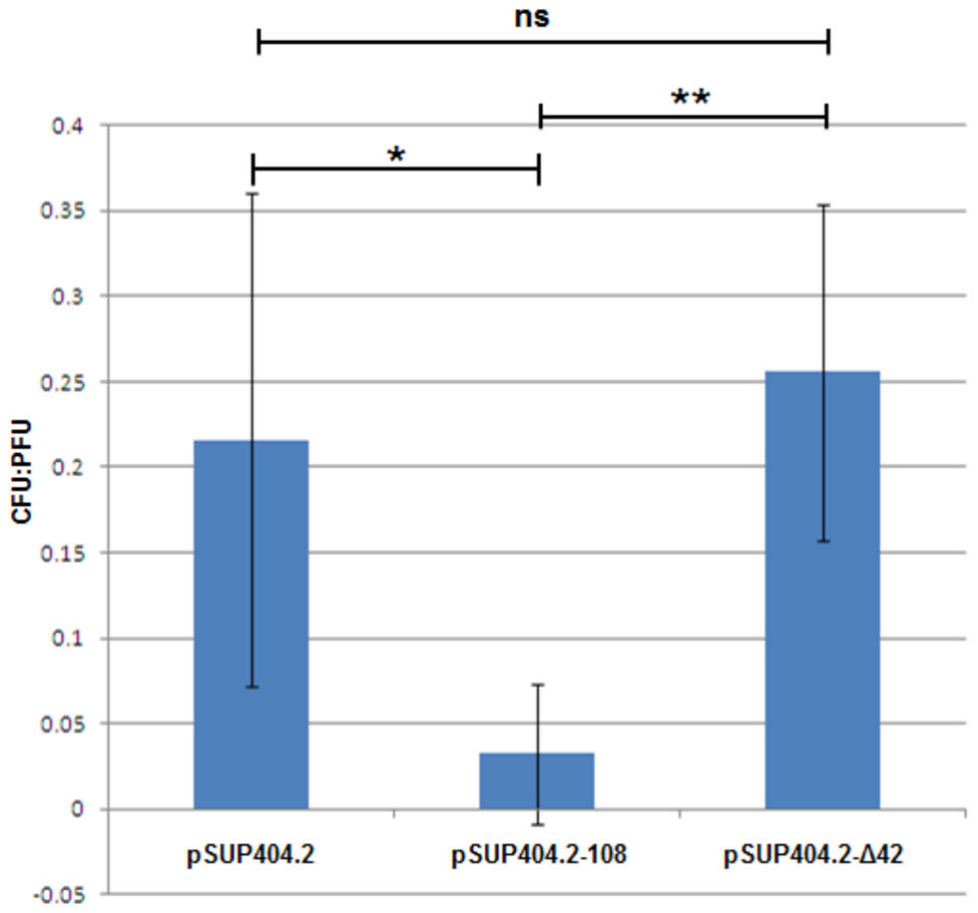
PFU to CFU ratio for pooled spontaneous HI strains and the markerless *bd0108* deletion HI mutants of *Bdellovibrio*. In the spontaneous generated HI strains; HID2 and HID26 (wild-type for *bd0108*) HID6, HID13, HID18, (point mutations in *bd0108*) and HID22 (*bd0108∆*42bp), and the markerless deletion mutants of *bd0108*, complementation tests shows that these strains carrying the pSUP404.2 plasmid encoding the wild-type *bd0108* is significantly deficient in the capacity to form HI colonies on PY agar plates compared to the capacity to form plaques on double layer overlay plates. This was significant compared to cells containing the pSUP404.2 plasmid alone, or the pSUP404.2-Δ42 with the 42 bp deletion variant of *bd0108*. The ratio of CFU:PFU was reduced from 2.16 x 10^-1^ in strains containing the pSUP404.2 alone to 3.21 x 10^-2^ in strains containing pSUP404.2-108 (p = 0.026) but were not significantly reduced relative to strains containing pSUP404.2-Δ42, 2.56 x 10^-1^ (p = 0.31). The difference between strains carrying the pSUP404.2-108 and the pSUP404.2-Δ42 was also significant (P<<0.01).

There was significant reduction in the number of colony forming units (CFU) on PY agar plates compared to plaque forming units (PFU) on overlayered prey-lawn plates. This was observed for all spontaneous and directed deletion *bd0108* mutants carrying pSUP404.2-108 compared to both pSUP404.2 alone, and pSUP404.2-Δ42 (Figure **4**). The ratio of CFU:PFU was reduced from 2.16 x 10^-1^ in strains containing the pSUP404.2 alone to 3.21 x 10^-2^ in strains containing pSUP404.2-108 (p = 0.026) but were not significantly reduced in strains containing pSUP404.2-Δ42; 2.56 x 10^-1^ (p = 0.31). The difference between strains carrying the pSUP404.2-108 and pSUP404.2-Δ42 was also significant (P<0.01). The results of this CFU:PFU assay indicates that expression of a wild-type copy of the *bd0108* gene inhibits the ability of *Bdellovibrio* to grow axenically as an HI culture to some extent in diverse *bd0108* mutant backgrounds, an observation in agreement with Roschanski and co-workers who observed that complementation with *bd0108* inhibited growth on autoclaved prey [12]. 

### Expression of bd0108 and bd0109 is from an operon including genes predicted to encode Type IVb pilus components

Expression of *bd0108* and *bd0109* was tested throughout the predatory lifecycle by analysis by RT-PCR on total RNA (from an equivalent starting number of *Bdellovibrio*) taken at various time points throughout the predation cycle, with only a small observable drop between the 15 minute time point (invasion) and the 30 minute time point (establishment) which agrees with previous transcriptional studies on *bd0108* (data not shown) [11,27].

**Figure 5:**
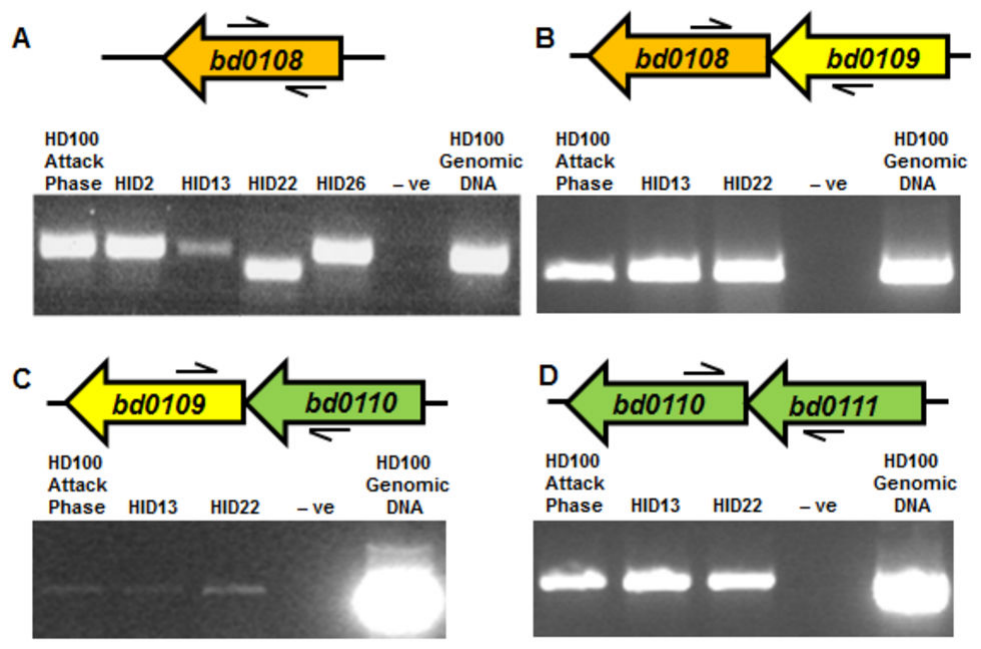
Expression of *bd0108* and co-transcription of surrounding *hit* locus genes using matched amounts of total RNA. In spontaneously generated HI strains; HID2, HID26 (with wild-type *bd0108*) HID13 andHID22 (*bd0108∆*42bp) there was transcription of *bd0108* (**A**). The primers flank the point of the 42 bp deletion in *bd0108* so for strain HID22 the PCR product is smaller. HID13, which has a mutated first codon (ATG->ATA) had lower amounts of expression of *bd0108* compared to other strains. RT-PCR analysis across the intergenic regions shows co-transcription of the gene pairs; *bd0108* and *bd0109* (**B**), *bd0109* and *bd110* (**C**), and *bd0110* and *bd0110* (**D**) in HI strains; HID13, HID22 and wild-type Attack Phase HD100. Primers are indicated by arrows above and below the gene cartoons.

We extended these studies using matched amounts of whole cell RNA from different HI and HD strains. RT-PCR analysis showed that in the spontaneous HI *bd0108* mutant isolates, expression of *bd0108* was still detectable, including in HID22 (*bd0108∆*42bp), although there was a difference in band size as the primers flank the region of the 42 bp deletion (Figure **5A**). Intriguingly, there was a slight, but reproducible, reduction in expression of *bd0108* in the HID13 strain (which has a point mutation in the ATG start codon) suggesting there might be a positive feedback mechanism on *bd0108* transcription that involves Bd0108 protein levels. 

Expression of *bd0108* and *bd0109* together, on the same mRNA strand, was also detected using RT-PCR analysis on RNA isolated from spontaneous HI strains with primers from gene *bd0108* to *bd0109*. Co-transcription was also observed for *bd0109*-*bd0110* and *bd0110*-*bd0111* (although a full length transcript from *bd0108-bd0111* was not detectable, possibly due to technical difficulties) suggesting that the putative type IVb pilus-related genes *bd0110*-*bd0111* are in an operon with *bd0108* and *bd0109* (**Figure 5BCD**)*.*

### Tryptophan fluorescence quenching and protease protection assays show interaction between Bd0108 and Bd0109

**Figure 6:**
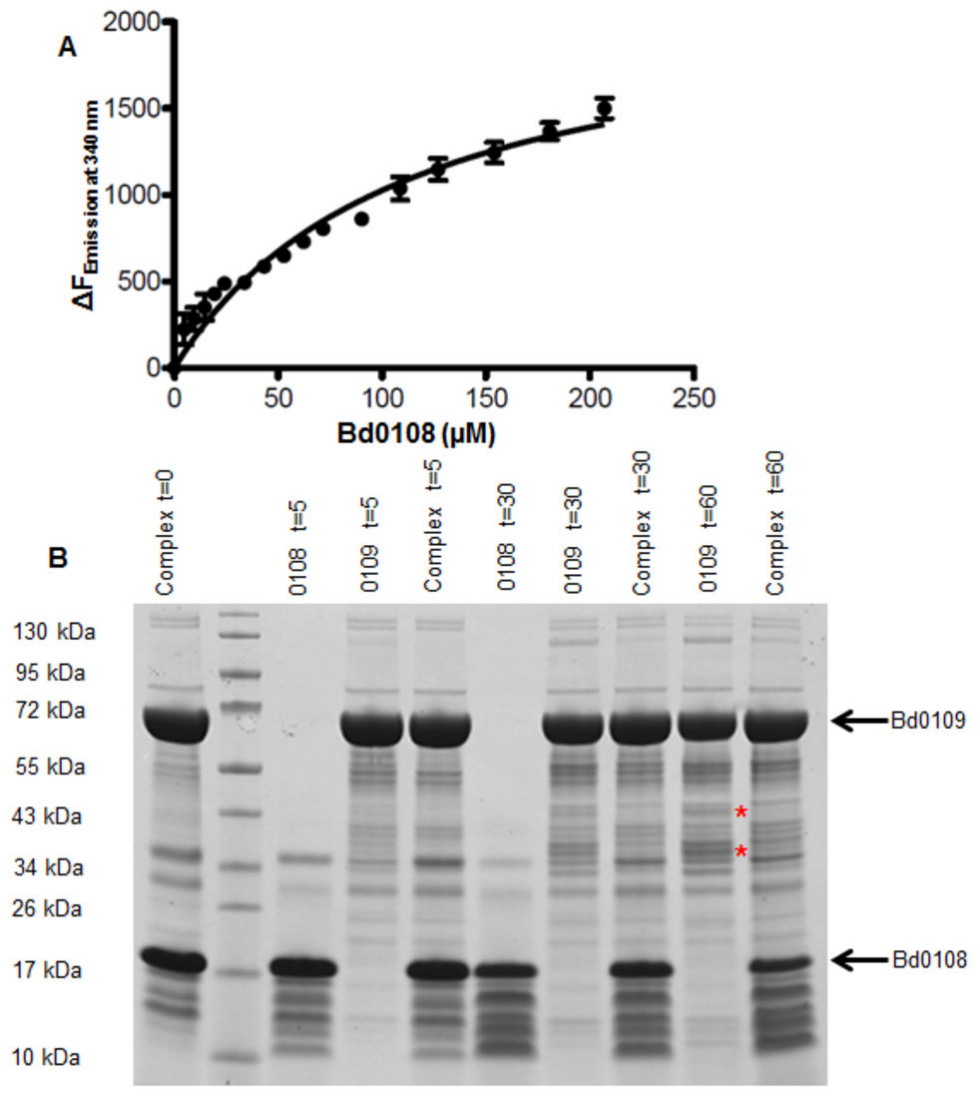
Evidence of interaction between Bd0108 and Bd0109. **A**. Fluorescence quenching assay of W fluorescence of Bd0109 by addition of (naturally non-fluorescent) Bd0108 protein. Bd0109 at a concentration of 2.41 µM was titrated with Bd0108 at 1.95 mM in non-reducing buffer. Increasing concentrations of Bd0108 resulted in increased quenching of fluorescence indicating interaction between the two proteins. **B**. Protease protection assay with Bd0109 and Bd0108 using chymotrypsin. The monomer of Bd0108 is around 17 kDa and the monomer of Bd0109 is around 65 kDa. Extra bands begin to appear around 35 and 43 kDa (indicated by asterisks) in the Bd0109 lanes treated with chymotrypsin which represent degradation products of Bd0109. These products cannot be seen in the lanes containing a complex of the two proteins (labelled complex) indicating that Bd0108 is interacting with Bd0109 to protect it from chymotrypsin digestion. Times indicated at the top are in minutes from adding the chymotrypsin.

As the *bd0108* and *bd0109* genes were co-transcribed, we postulated that they might work together in regulating the switch to HI growth by *Bdellovibrio*. Thus both genes were expressed and the proteins they encoded were purified. The natural tryptophan content of Bd0109 and the absence of tryptophan in Bd0108 allowed us to study the interactions of the purified gene products of the two adjacent *hit* locus genes (Figure **6**). It was clear that addition of Bd0108 protein quenched the fluorescence of Bd0109 showing that the two proteins interact (Figure **6A**). Also interaction of the proteins before the administration of the protease chymotrypsin protected Bd0109 against proteolytic digest (Figure **6B**). Thus we propose that Bd0109 and Bd0108 interact *in vivo*. As both proteins have predicted N terminal secretion signals (see below) this interaction could take place in the *Bdellovibrio* periplasm or externally. 

### Bioinformatic analysis suggests a possible pilus-regulation role for Bd0109

BLAST analysis of the Bd0109 sequence reveals homologies to wall-associated protein WapA, YD-repeat containing proteins and RHS elements. All of these classes of protein contain the PFAM RHS repeat (PF05593) which consists of a repeating YD element originally named RHS for Recombination HotSpot [28,29] and thought to be involved at the protein level in sugar binding [30]. These proteins are a diverse group [31] with ill-defined or diverse functions, possibly a result of the proteins having a conserved YD-repeat core domain with differentiated N- and C- terminal domains. Two reports [32,33] have identified RHS elements as having a toxin/antitoxin role with a smaller downstream protein acting as the antitoxin. One report has shown an rhs mutant to be defective in bacteriocin production [34], although the mechanism for this is unclear. Another report [35] assigns a role for RhsA in coupling capsule synthesis with export in *E. coli*. A further report links RHS function to pili by showing that in *Myxococcus* [36], a mutant of a YD-repeat protein no longer retracts pili in the presence of methylcellulose. 

**Figure 7:**
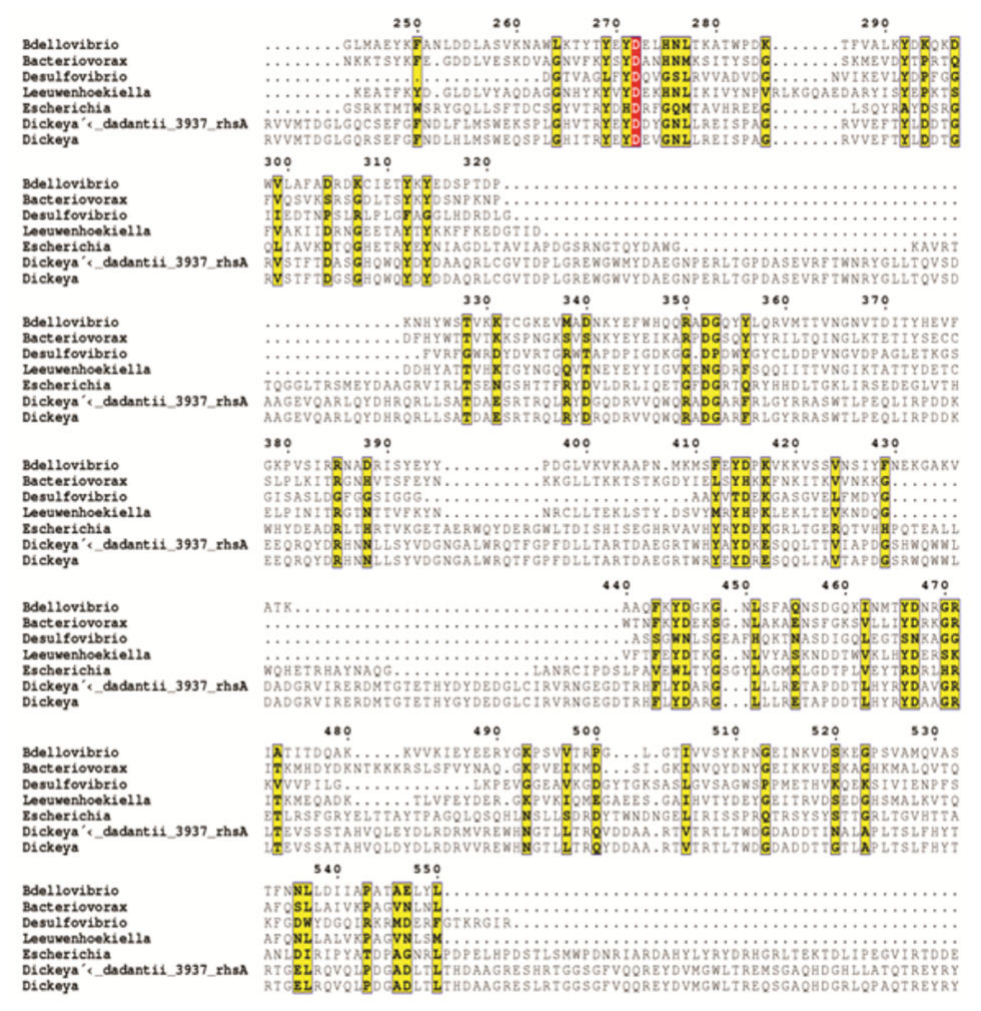
Alignment of Bd0109 with other RHS elements. Multiple sequence alignment constructed using Clustal W showing that the main regions of homology between the *Bdellovibrio* Bd0109 predicted protein and other RHS elements is the core pFAM RHS repeat (PF05593) region which consists of a repeating YD element. The whole Bd0109 sequence contains 13 YD or YE sequences and several other Y residues. The sequences aligned are from the genera indicated with the names, with the second *Dickeya* sequence being RhsB.

Taken together, the work so far on RHS elements suggest that the common attribute may be a sugar binding YD-repeat region (of widely varying size due to different numbers of repeats) with variable N- and C- termini which could confer a wide variety of functions. There are no determined structures for a member of the YD-repeat family; however, the (predicted) β-rich architecture and carbohydrate binding function mirror that of the wider beta-solenoid grouping e.g. toxin A of *Clostridium difficile* [37] or the LytA cell-wall binding protein [38]. Multiple sequence alignment (Figure **7**) shows that the majority of sequence similarity of Bd0109 with RHS elements is to the YD-repeat core. 

Analyses using BLAST [39], SMART [40] and STRING [41], identify four other RHS elements in the *Bdellovibrio bacteriovorus* HD100 genome and two of these are also next to pilus-associated genes: *bd1292* is proximal to *bd1290 pilA* and *bd1291 pilG*; *bd3309* is near *bd3306 pilQ* and *bd3307 cpaF*. The other predicted RHS element *bd0328* and *bd2719* are not proximal to obvious pilus associated genes. Interestingly, a small region of the “variable” N-terminal domain of Bd0109 (the YD repeats are clustered toward the middle and C-terminus of the protein) has a potential agreement with a pilin fold, as judged by threading using the PHYRE server [42], amino acids 20-63 of Bd0109 aligning with residues 233-281 of *E. coli* PapGii (28% sequence identity; PDB code 2wmp). Thus bioinformatically Bd0109, (which we have shown experimentally to interact with Bd0108) is likely to have a pilus-associated interaction and a cell wall binding phenotype. 

### The ∆Bd0108 and Bd0108∆42bp mutants display two differing piliation phenotypes that deviate from wild-type strains

**Figure 8:**
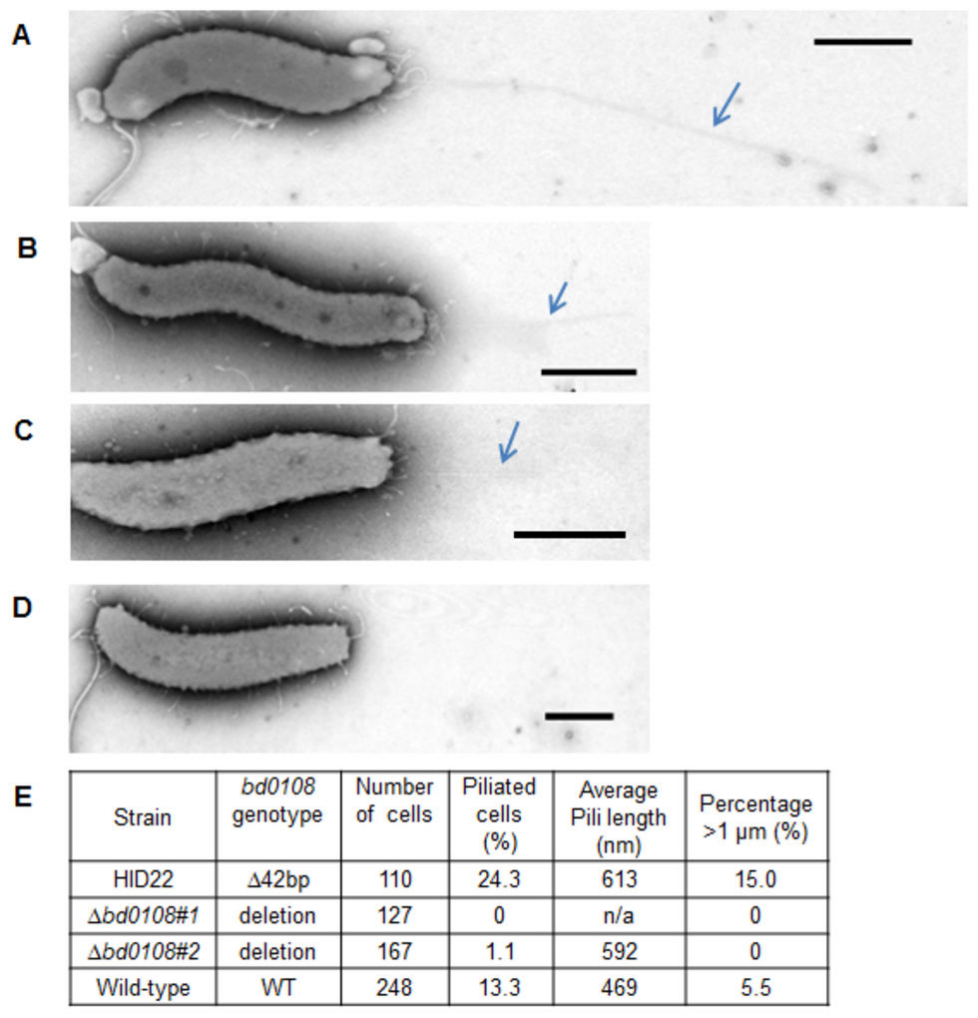
Electron micrographs of HI *Bdellovibrio* showing the presence and absence of cell surface structures. Representative electron micrographs of cells stained with 2% PTA pH7.0 to show long straight pili structures. **A**) An example of a HID22 (*bd0108∆*42bp) cell showing a pilus greater than 1 µm that occurred in 15% of the isolates. Average representatives of HID22 cells (**B**), and of a HI strain containing a wild-type *bd0108* gene (**C**). The Δ*bd0108* markerless deletion strain showing no pilus-like structures (**D**). **E**) A table showing 4 HI isolates and their average presence/absence and length of pili. Scale bars = 500 nm.

In order to test if Bd0108 (and by inference Bd0109) were associated with pilus function, both the spontaneous and directed *bd0108* HI mutants were examined by electron microscopy (Figure **8**). Strains HID2 and HID26, which had no mutations in *bd0108*, had 1-4 pili per cell with varying lengths, but only a percentage of cells (13 ± 3.3%) had any pili visibly extruded. The vast majority (>95%) were seen at the non-flagellated pole although some were seen at the flagellated pole, there were also rare incidences of pili being present at both poles of the same cell (data not shown). This compares with a previous study [1] where strain HID2 (wild-type for *bd0108*) had been analysed alongside wild-type, host dependent strain HD100 and revealed that a small percentage of wild-type cells showed visibly extruded pili. By contrast, the *bd0108* deletion strains had almost no visible pili extruded, with pili observed on only 2 cells of 392 (0.005%) from the two strains. Unexpectedly, the HID22 (*bd0108*∆42bp) strain had a higher percentage (24.3%) of piliated cells than the wild-type (p=0.056). Extra-long extruded pili (over 1 µm in length) were visible on the HID22 (*bd0108*∆42bp) strain more frequently than for wild-type *Bdellovibrio* or the directed *bd0108* deletion mutants (15% having pili >1 µm, compared to 5.5% for wild-type)*.*

Cells from these HI strains were concentrated by low speed centifugation, matched by OD_600_, and then subjected to shearing [43] in order to detach and analyse the pili. The supernatants were spun in an ultracentrifuge and the recovered pellet was analysed by Tricine SDS-PAGE [44]. The HID22 (*bd0108∆*42bp) sample showed two protein bands (Figure **S2**) at higher concentrations than in the other *Bdellovibrio* HI strains at around 17 KDa and 20 KDa. Mass spectrometric analysis showed that the 20 KDa band contained PilA (Bd1290) protein indicating that the extruded pili seen in HID22 (*bd0108∆*42bp) were likely to be Type IVa pili. No other types of pilin were detected in these analyses of HI cells; the 17 KDa band contained degraded fragments of flagellins and *Bdellovibrio* hypothetical proteins, none of which had homology to pilins. The 20 KDa PilA band was not detectable in the sheared extract from Δ*bd0108* HI isolates, corresponding to the almost complete absence of pili seen by electron micrographs.

### Complementation with wild-type *bd0108* increases levels of piliation in HD *Bdellovibrio*

It was not possible to test complementation of the pilus phenotype in the HI grown mutant strains, as the presence of extra copies of *Bd0108* suppressed HI growth as discussed above (Figure **4**). Instead, complementation was attempted by growing the plasmid-bearing cells host-dependently (HD). The HD cells contained the replicating plasmid pSUP404.2 with and without *bd0108* (and 200 bp of flanking DNA to include the promoter). In these HD conditions, the overall level of piliation was greater (Table **2**), but a very significantly higher percentage of piliated cells were seen under conditions where plasmid borne *bd0108* was present. These pili were also longer on average than wild-type (P<0.01) and cells had a higher percentage of highly extruded pili longer than 1 µm. This confirms that the presence of a wild-type copy of *bd0108* is involved in up-regulating the extrusion of pili. 

**Table 2:** Number and length of pili when the *∆bd0108* deletion strains were grown host dependently with and without complementation by the plasmid pSUP404.2 containing a wild-type copy of *bd0108* with flanking DNA.

**HD *Bdellovibrio* Strain**	**Number of cells**	**% of cells piliated**	**Average length of pili (nm)**	**Percentage over 1 µm**
∆*bd0108#1* (pSUP404.2)	73	12.3	245	0
∆*bd0108#2* (pSUP404.2)	91	13.2	201	0
∆*bd0108#1* (pSUP404.2-108)	56	39.3	541	22.6
∆*bd0108#*2 (pSUP404.2-108)	69	42.0	402	8.7

The complemented strains had more pili with a longer average length showing that the paucity of pili in the mutants was complemented (P<0.01).

### C-terminally fluorescently tagged Bd0108, Bd0108∆42bp and Bd0109 were not visibly fluorescent in *Bdellovibrio* but located in the *E. coli* periplasm when heterologously expressed

To try to determine the cellular locations, C-terminal fusions of the Bd0108 protein and the 42 bp deletion variant with an mCherry fluorescent protein, and a Bd0109 fusion with an mTFP fluorescent protein were made in both wild-type *Bdellovibrio* and the markerless *bd0108* deletion mutant. The fusion constructs were designed to integrate into the *Bdellovibrio* genome by a single crossover into the targeted gene such that the fused gene would utilise the natural promoter of the *bd0108*/*bd0109* gene, as used successfully for other genes in *Bdellovibrio* [45]. Despite successful integration of the construct into the expected target gene, fluorescence was not observed in any of the resulting *Bdellovibrio* cells. This may be due to some autoregulatory effect of the tagged proteins on their own expression, possibly associated with a failure to export the proteins due to the tag.

**Figure 9:**
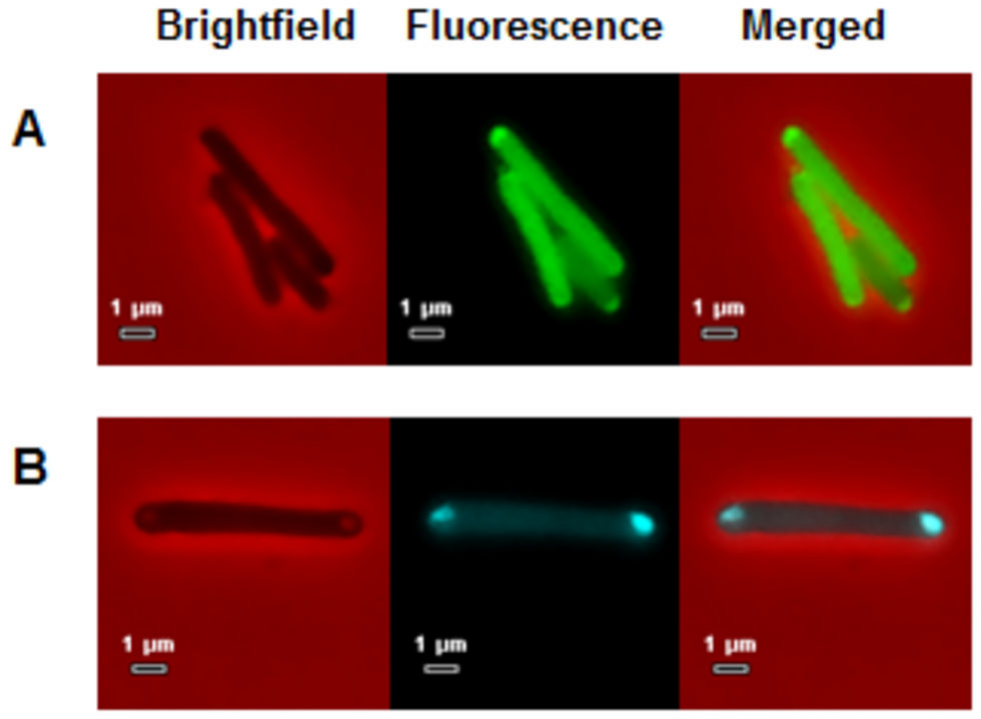
*In trans* expression of fluorescently tagged Bd0108 and Bd0109 in *E. coli* S17-1 heterologous host. **A**. *E. coli* S17-1 expressing a C-terminal mCherry fusion of Bd0108 protein *in* *trans*. Fluorescence can be seen in the periplasm of the cells, with large amounts towards the poles of the cell where cells are plasmolysed. **B**. *E. coli* S17-1 expressing a C-terminal mTFP fusion of Bd0109 protein *in* *trans*. Fluorescence can be seen in the periplasm of the cells, with large amounts towards the poles of the cell where cells are plasmolysed.

However fluorescence of both the Bd0108mCherry and Bd0109mTFP in the heterologous host *E. coli* strain S17-1 was observed (Figure **9**), for the same plasmids that had been conjugated into the *Bdellovibrio*. For all of the tagged proteins, the fluorescence was located in the periplasm of *E. coli* as fluorescence was seen throughout the cell, but was seen greatly concentrated in polar regions of some cells which appeared to be plasmolysed (Figure **9**), indicating a periplasmic location [46,47]. (This was also the case for the Bd0108∆42bp-mCherry tag - data not shown). Sequence analysis using SignalP (http://www.cbs.dtu.dk/services/SignalP) of the amino acid sequence of Bd0108 suggests there is a secretion sequence in the first 23 amino acids with a cleavage point between the 23^rd^ and 24^th^ residue (ASA-DE) (C-score of 0.859). SignalP also predicts a secretion sequence in Bd0109 with a cleavage point between the 18^th^ and 19^th^ amino acid residue (AFA-LV) (C-score of 0.902), so it is possible that both of these proteins are targeted to the Sec pathway of *E. coli* [48] which results in the periplasmic location observed. Although this could not be proved by microscopy directly in *Bdellovibrio*, this evidence predicts that the native Bd0108-0109 proteins are targeted to the periplasm.

### RNA-seq analysis of transcription in HI growth conditions

As our data suggested that Bd0108 (possibly via interaction with Bd0109) may be involved in a switch between HI growth and predatory growth, we examined the global transcription pattern of HI mutants with *bd0108* deletions or point mutations, versus predatory HD100 cells, to attempt to find clues as to how this switch was being implemented. RNA-seq was chosen as this would give us insights into regions of the genome not previously examined in array studies, such as small RNAs. In order to validate the new method, we used samples of host dependent, attack phase HD100 and host independent HID13 which we had previously analysed by arrays [49], and prepared RNA samples in the same way (by growing HI cells to mid-log phase). 

In our comparison of these vastly different lifestyles of *Bdellovibrio*, in a previous published study, our statistical analyses of the arrays had shown 1559 genes to be significantly upregulated and 1319 downregulated in the switch between attack phase and HI growth [49]. In this current study, by choosing genes differentially regulated by 2-fold or more, the RNA-seq dataset generated matched the array results reasonably well: 1352 genes upregulated (overlap of 1188 with the array dataset) and 1421 downregulated (overlap of 788 with the array dataset) in the switch between attack phase and HI growth. Other comparisons have validated RNA-seq results by comparison to previously published array results and found similar overlap between the techniques [50]. As well as the different analysis methods, the differences can be attributed to intergenic regions not represented in the arrays (45 in the upregulated dataset and 138 in the dowregulated dataset) and the inherent differences in the techniques (e.g. misrepresentation by primer design of the arrays and some cross reaction of the arrays by small amounts of remaining prey RNA). The other samples analysed (grown axenically to mid-log and matched by OD_600_, in order to compare between HI datasets) were the ∆*bd0108* strain and HID22 (*bd0108∆*42bp), which had given the varied phenotypes described above. 

The first observation from these analyses is that there are a large set of common HI genes differentially regulated relative to the HD attack phase; 1163 upregulated and 1207 downregulated in all three HI strains. The overlap with the array datasets (830 upregulated common genes, by both array and RNA seq, and 594 downregulated) confirms that the conclusions drawn from these are universal to HI growth and not just an artefact of an individual HI strain studied [49]. This is an important finding as HI strains vary considerably in many phenotypes observed such as growth rate, morphology and cell colouration [51]. Because of these phenotypic variations, it is perhaps unsurprising that there were also some differences between transcriptional profiles of the HI strains shown in Table **3** below. Datasets of differentially regulated genes which were unique to each strain were analysed to give indications of mechanisms of the different phenotypes of the strains. 

**Table 3:** Differentially regulated genes datasets for each of the HI strains showing the number of genes differentially regulated between the attack phase and HI growth phase.

Strain	Number of genes/intergenic regions differentially regulated >2-fold			
	Upregulated vs HD100	Downregulated vs HD100	Upregulated vs HD100, unique to this strain	Downregulated vs HD100, unique to this strain
HID13	1352	1421	44	43
HID22	1333	1422	82	113
∆*bd0108*	1420	1463	74	55
Common to all HI strains	1163	1207	N/A	N/A

HID22 *Bdellovibrio* cells (*bd0108∆*42bp) had often longer and more frequently observed pili than the other HI strains (Figure **8**). Overexpression of wild-type *bd0108* upregulates the number of extruded pili. Despite the phenotype of the 42 bp deletion strain; the majority of pilus-related genes are in the RNA-seq dataset of genes transcriptionally downregulated in **all** HI strains relative to highly piliated HD100 attack phase. These include the operons and genes: *bd0108-0121, bd0470 tadC, bd0793* and *bd3307 cpaF* (Type IVb genes) and the Type IVa structural genes *bd1509-1512, bd0867 pilQ, bd1290 pilA, bd1585 pilM, bd2167 pilL*, and *bd3852 pilT*. We did find that many of these pilus genes were downregulated less in HID22 (*bd0108∆*42bp) compared to the *bd0108* deletion strain and HID13 (spontaneous HI *bd0108* ATG-ATA start codon point mutant). Transcriptional differences seem unlikely to be the explanation of this excessive extrusion phenotype, as the pilus genes are still downregulated in HID22 (*bd0108∆*42bp). Moreover, pilus assembly tends to be post-translationally regulated at the stage of pilus fibre assembly; ready-made PilA protein subunits are exported from the inner membrane during extrusion, to form new pilus fibres [52].

As complementation of ∆*bd0108* mutants with the full- length *bd0108* gene suppressed HI colony growth more than with the ∆42bp version of *bd0108*, we interrogated the datasets for any indications of differences in the way that the switch to HI growth was being regulated in *Bdellovibrio* with the total deletion *of bd0108* versus the ∆42bp*bd0108*. Table **4** lists the potential regulatory genes uniquely differentially regulated for each HI strain suggesting that they may use different regulation pathways for HI growth. Although there are too many differentially regulated genes to identify specific pathways, these data give indications for future investigations. Each strain also has different intergenic regions where transcripts were differentially regulated versus HD100 attack phase (2 upregulated and 12 downregulated in HID13; 4 upregulated and 15 downregulated in HID22 (*bd0108∆*42bp); 16 upregulated and 6 downregulated in the *bd0108* deletion strain). This is the first time intergenic regions representing non-coding RNAs have been reported in *Bdellovibrio* and the numbers differentially regulated suggest that they may have significant roles in regulation. 

**Table 4:** List of genes differentially regulated in each HI strain with products predicted to have regulatory functions.

**Potential regulatory genes differentially regulated >2-fold only in HID13 vs HD100**			
**Bd number**	**Gene**	**Product**	**Log_2_ fold regulated**
Bd1394	*dbpA*	ATP-dependent RNA helicase DbpA	1.073183
Bd3649	*bd3649*	response regulator	1.063815
Bd0242	*rpoD*	RNA polymerase sigma factor RpoD	-1.03966
Bd1657	*bd1657*	sensory box histidine kinase	-1.04372
Bd2300	*xerD*	integrase/recombinase XerD	-1.4758
**Potential regulatory genes differentially regulated >2-fold only in HID22 vs HD100**			
Bd0300	*bd0300*	sensory transduction histidine kinase	1.223213
Bd0561	*rsbU*	sigmaB regulation protein RsbU	1.058021
Bd1017	*phoB*	DNA-binding response regulator PhoB	1.13589
Bd2426	*bd2426*	rhomboid-like protein (RRP)	1.642479
Bd3229	*bd3229*	LysR family transcriptional regulator	1.755906
Bd3393	*ragB*	RagB (two-component sensor histidine kinase)	1.128933
Bd3521	*bd3521*	TetR family transcriptional regulator	1.904378
Bd3613	*bd3613*	sensory transduction histidine kinase	3.57316
Bd3677	*paiB*	transcriptional regulator protein Pai2	1.258287
Bd0811	*araC*	AraC family transcriptional regulator	-1.96659
Bd0914	*bd0914*	DNA polymerase epsilon subunit	-1.37525
Bd1127	*bd1127*	LysR family transcriptional regulator	-1.2427
Bd2205	*sulA*	cell division inhibitor SULA	-2.09035
**Potential regulatory genes differentially regulated >2-fold only in ∆*bd0108* vs HD100**			
Bd0711	*bd0711*	DNA-binding protein HU	1.550853
Bd0897	*bd0897*	LysR family transcriptional regulator	1.041405
Bd1002	*soxR*	redox-sensing activator of soxS	1.120062
Bd1759	*kdpE*	two-component response regulator KdpE	1.173304
Bd1820	*bd1820*	poly A polymerase	1.076493

### A small, non-coding RNA between *bd0103* and *bd0108* is upregulated in all *bd0108* mutants compared to HID2 and HD100 strains with a wild-type *bd0108* gene

**Figure 10:**
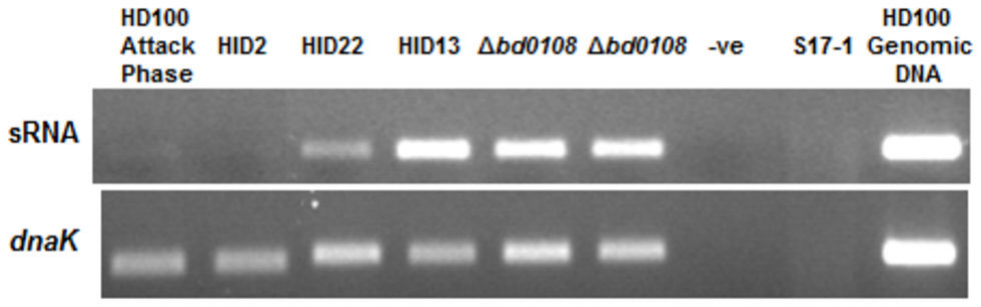
RT-PCR expression of the sRNA downstream of *bd0108* and expression of a control gene *dnaK*. Expression of a small, non-coding RNA downstream of *bd0108* was shown to be different in HI strains carrying different mutations in *bd0108* and attack phase HD100. Expression was highest in HID13 (ATA->ATG start codon mutation) and the strains with the markerless deletion of *bd0108*. Strain HID22 (*bd0108∆*42bp), had slightly lower expression, while those strains with a wild-type *bd0108*; HD100 and HID2, show much lower expression of the sRNA. Expression of *dnaK* was uniform across the samples indicating a matched amount of total RNA was used for the experiment.

RNA-seq analysis identified a small non-coding RNA located between the transcriptional units of *bd0103* and *bd0108* (bases 96436-96822 of HD100). Manual inspection of the intergenic region suggested the presence of a sRNA-encoding gene between positions 96455 and 96716 (261 bp). RT-PCR analysis using primers designed to anneal to this RNA shows that in all HI strains, this was significantly up-regulated relative to the HD100 attack phase and to strain HID2 which each have a wild-type *bd0108* gene (Figure **10**). In the HID22 (*bd0108∆*42bp) strain the level of upregulation was slightly, but repeatably, lower than in the *∆bd0108* strains and in the HID13 strain which has a start codon point mutation in the *bd0108* gene. This fits with the hypothesis that the small non-coding RNA is regulated in association with *bd0108* expression.

## Discussion

The transition from the replication arrested “attack-phase” state of HD *Bdellovibrio bacteriovorus* to the replication-competent, HI, axenic growth state was previously found to be firmly associated with, but not absolutely requiring, mutations in a small *Bdellovibrio*-specific gene *bd0108*. This was shown by identification of point mutations both in local and genome wide studies by Cotter and Thomashow [8], Roschanski and co-workers [12] and the Jurkevitch group [51]. It was noted [10,11] that the *bd0108* gene lies adjacent to a cluster of Type IVb pilus associated genes. We have shown that there is co-transcription of gene *bd0108* along with *bd0109*, and also co-transcription of *bd0109* with adjacent Type IVb pilus associated genes. This suggests that there may be shared and non-shared promoters for these genes and that they are co-transcribed with genes from the pilus operon at least to some extent.

It was previously reported that diverse point mutations and an in frame 42 bp deletion and truncation of the *bd0108* gene were all associated with axenic growth in *Bdellovibrio* and also associated with a drop in predatory efficiency [9,12]. Despite this, axenically growing *Bdellovibrio* were isolated that did not have *bd0108* mutations; genome wide DNA sequencing studies of Wurtzel and of Roschanski found mutations elsewhere in the genome. Genes associated with RNA processing were the only category of non-*bd0108* mutations found commonly in the HI strains (five strains in total between the two studies and different genes involved in RNA processing). These interesting findings suggested a regulatory role for the product of gene *bd0108*, but a role that was possibly reliant on “licensing” by another physiological state of the *Bdellovibrio*. Thus our first hypothesis was that the majority of *Bdellovibrio* had to have *bd0108* point mutations to grow axenically, because the physiological state at which they were “offered” artificial growth media required them to curtail a predatorily-associated process that blocks entry into axenic growth. 

Previous investigations [10,11] of the *hit* locus had pointed towards a possible association with Type IVb pilus genes. Because previous studies by ourselves [1] and confirmed by others [2] had shown that expression of a Type IVa pilus fibre was required for prey entry by predatory *Bdellovibrio*, we hypothesised that the “licensing” state could be associated with pilus extrusion. We knew from our microscopic studies of pili on wild-type HD *Bdellovibrio* [1] that pilus extrusion is far from 100% in populations of *Bdellovibrio* with wild-type pilus genes and thus (similarly to the situation in other bacteria) pilus extrusion is probably subject to complex cues. Finding that *Bdellovibrio* strains with a full deletion in gene *bd0108* produced only very few visible pilus fibres (Figure **8**) and no detectable protein band corresponding to Type IVa pilus fibre Bd1290 PilA (Figure **S2**) showed that Bd0108 has a role in pilus fibre regulation.

Complementation of Δ*bd0108* in attack phase cells results in more frequently extruded and longer pilus fibres (Table **2**), which confirms a role in pilus extrusion for *bd0108*. So our interim conclusion was that a paucity of Type IVa pili, caused by deletion of *bd0108*, would promote axenic growth (concomitantly diminishing predatory growth of the population). However we were surprised to find that our directed whole Δ*bd0108* strain could still prey upon other bacteria- albeit at a lower efficiency than when it was complemented with wild-type gene *bd0108*. This was something that Roschanski and co-workers had also reported for their truncated *bd0108* transposon mutant [12]. Because we knew [1,2] that a Type IVa pilus was essential for prey entry, we thoroughly examined the outer surface of the ∆*bd0108* cells in case there was an alternative pilus (for example Type IVb) being expressed to allow prey entry. There was not, although we acknowledge that due to the tight association between predator and prey it was not possible to discern any pilus expression at the point of prey invasion. 

During this examination, we found the surprising result that the HID22 (*bd0108∆*42bp) strain did not show the same pilus phenotype as the ∆*bd0108* strain. Indeed they showed more frequent pilus extrusion, a higher frequency of longer pili and a stronger band containing PilA protein in surface sheared extracts than did the wild-type *Bdellovibrio* with no *bd0108* point mutation. Thus we propose that the 42 bp deletion form of Bd0108 produces pili that are more frequently extruded and which cannot be as efficiently retracted into the cell. It will take further substantial experimentation, beyond the scope of this paper, to study pilus retraction in *Bdellovibrio* (as this is challenging in all bacteria). However, we conclude from our microscopy (Figure **8**) that having very fewer extruded pili causes a similar signal, promoting axenic HI growth to the *Bdellovibrio*, as extruded and less frequently retracted pili.

Having established a role for Bd0108 in pilus formation, we turned to the mechanism of action. Nothing in the predicted structure of the *bd0108* gene product suggested that it is a typical part of a pilus complex and as we had found that there was a shared transcript for *bd0108* and *bd0109*, we hypothesised that Bd0109 might be part of such a regulatory system. Both proteins are predicted to be exported across the cytoplasmic membrane, due to N-terminal signal sequences. Fluorescently tagged Bd0108 and Bd0109 gave no expression in *Bdellovibrio* itself, but we found that when heterologously expressed in *E. coli* (which does not have Type IV pili), both were strongly expressed in a periplasmic location (Figure **9**). This suggested that Bd0108 and Bd0109 are normally periplasmic, but the fluorescent tag prevents export through pathway(s) in *Bdellovibrio* that may be different to the (probably Sec) pathway they were exported by in *E. coli.*

Although we could predict, rather than prove, a periplasmic address for Bd0108 and Bd0109 in *Bdellovibrio*, it was striking (Figure **7**) that the Bd0109 structure contains a repetitive YD motif which is found in proteins that interact with carbohydrate polymers (as found in the bacterial cell wall; homologues of this protein WapA are wall associated in *Bacillus* [53]). Our results from native tryptophan fluorescence quenching studies and chymotrypsin protection assay (Figure **6**) indicate that the Bd0109 and Bd0108 proteins interact with each other. These findings suggest that Bd0108 and Bd0109 work as a regulatory complex at a cell wall location, in the periplasm, to control pilus extrusion. Without the Bd0108 protein in the complex very few pili are extruded, but with the mutant form of the Bd0108 protein (encoded by the 42 bp deletion form of the gene) in the complex, pilus extrusion/retraction is deregulated and more pilus extrusion, compared to wild-type, occurs (and/or pili are retracted less). 

This hypothesis is supported by observations that a YD-repeat protein null mutant in another deltaproteobacterium, *Myxococcus xanthus*, was defective in pilus-driven motility, as it could not retract the pilus to pull the cell along, and this mutation was not complemented by addition of methylcellulose (which normally stimulates pilus retraction in those bacteria) [36]

Amino-acids 20-63 of Bd0109 have a potential pilin fold, as judged by threading using the PHYRE server, so it is possible that this domain interacts directly with the growing pilus fibre to change its retraction or extrusion in a way analogous to that of minor pilins [54]. Type IV pili are associated with many varied functions in different bacteria, several of which are related to retraction and extrusion [55] and it is possible that YD-repeat proteins are involved in regulating this process in other pilus systems. Other phenotypes of YD-domain proteins include deficiencies in export [34,35] which also may be pilus-mediated. It is therefore possible that we have discovered in *Bdellovibrio* a system of pilus regulation that may have analogues in other non-predatory bacteria.

Because directed deletion studies for *bd0109* yielded no mutants, despite screening numbers of cells many times more than was required to isolate a *bd0108* deletion (Table **1**; and deletion of many other genes in *Bdellovibrio* [13,45]); and because no spontaneous mutations associated with axenic growth have ever been reported in *bd0109*, we propose that the role of Bd0109, possibly at the cell wall-pilus fibre interface, may be essential to provide a positive signal for *Bdellovibrio* viability or growth/replication. We conclude that Bd0108 is a modifier of the activity of Bd0109 and that parts (but not all) of its interaction faces with Bd0109 include amino-acids normally encoded by the 42 bp of gene *bd0108*. These amino acids are frequently deleted in axenic *Bdellovibrio* strains. Changing the Bd0108-Bd0109 interaction, by *hit* locus mutations in *bd0108*, promotes HI growth, and there is a signal sent that involves/monitors pilus extrusion/retraction status. It may be that activity at the pilus extrusion/retraction motor sends the signal for *Bdellovibrio* growth or that Bd0108 occupancy on Bd0109 sends the signal via interaction with as yet undiscovered proteins.

There is some evidence for the pilus-specific signalling proposal; Vik and co-workers have recently reported a pilus associated requirement for bacterial growth in *Neisseria* [56]. It is possible that the Bd0109-0108 protein complex is part of such a regulatory system where the Type IVa pilus extrusion/retraction activity “reports” the environment back to the cell, determining whether it grows or not. It is clear in the *Bdellovibrio* system that Bd0108 is a more unusual modifying component of such a system, specific to *Bdellovibrio*, but Bd0109 does have homologues in other bacteria and those Bd0109 homologues are associated with the Tad/Type IVb pili of other bacteria.

We postulate that in *Bdellovibrio* this regulatory system has become associated with Type IVa pili instead. Rendulic and co-workers [10] did report the apparent absence of Type IVb pilus fibre genes in the *Bdellovibrio* genome, reporting on the *hit* locus pilus genes as being a partial set of Type IVb pilus genes. Schwudke and co-workers reported that Bd0118 and Bd0119 had regions with some similarity to flp pilins, but did not detect any of these proteins in fractions that contained PilA protein [11]. Type IVa pili are required for prey invasion in *Bdellovibrio*, a novel activity that is not found for these pili in other bacteria [1,2]. Prey invasion, likely concomitant with Type IVa pilus extrusion/retraction, brings with it the switch from non-replicative attack phase life, (hunting outside prey cells) to replicative growth within prey. Thus pilus retraction, regulated or monitored by the Bd0109-Bd0108 complex (likely at the *Bdellovibrio* cell wall), is a process which controls the switch between non-replicative predatory hunting and axenic replication in high nutrient situations (in prey or artificial media). 

RNA-seq analysis of HI strains with diverse *bd0108* genotypes revealed a large, common subset of genes differentially regulated between HI growth phase and attack phase cells. Significantly, this validates previous results from our array analyses [49] which compared a single HI strain and shows that, despite a diversity of morphological and septation phenotypes observed between HI strains, there are a large set of core processes common to all. The vast majority of genes involved in pilus formation were similarly downregulated considerably from attack phase to HI growth phase in all of the HI strains with differing piliation. This indicated that pilus phenotypes were unlikely to be caused at a transcriptional level, agreeing with the fact that pilus-fibre formation is subject to post-translational control. The fact that each HI strain had a significant number of differentially transcribed genes (Table **3**) including predicted regulators (Table **4**) indicates that HI strains with varying *bd0108* mutations may control HI growth in differing ways. There are two results which support this idea: 1) complementation with *bd0108*, but not with the 42 bp deletion form suppressed HI growth (Figures **3** and **4**) and 2) a point mutation in the ATG (strain HID13) results in lower expression of *bd0108* (Figure **5**). This may account for the varying morphological and septation phenotypes seen amongst different HI strains as different regulatory networks are turned on or off. 

The nature of the signalling pathway(s) from pilus extrusion/retraction to transcriptional regulation for HI or intraperiplasmic growth could be investigated by selective gene deletions from the differentially regulated datasets in extensive future work. It is clear that Bd0108 forms a complex with Bd0109 and our hypothesis is that this periplasmic complex (possibly binding at the cell wall) regulates pilus extrusion/retraction activity. In predatory invasion, changes in the pilus extrusion/retraction state, caused by the pilus binding and unbinding a substrate, which may be the prey cell outer surface, results in a signal which switches attack phase cells into growth. Recently Cahoon and Siefert [57] have shown the involvement of sRNA signalling in pilus antigenic variation in *Neisseria* and Lui and co-workers demonstrated sRNA regulation of pilus expression and cell adhesion [58].

In that context, our discovery that a 261 bp small RNA is specifically upregulated in the strains with *bd0108* mutations, but not in wild-type HI strains HID2 and HID26 or in HD100 (Figure **10**) may be informative as to a mechanism. It is possible that this may indicate the nature of a sRNA regulatory pathway that toggles on and off replication initiation. Thus the axenic growth preponderance shown by the *bd0108* mutant strains could be controlled by a sRNA-dependent pathway. The essentiality of the Bd0109 protein for viability of *Bdellovibrio* (Table **1**) may be further evidence for the link between that sensory pathway and cell replication. Access-based target prediction [59] for the *Bdellovibrio* sRNA resulted in top targets of hypothetical proteins, so there is not yet an obvious pathway to test for this, but mutation of relevant genes may elucidate this in future.

**Figure 11:**
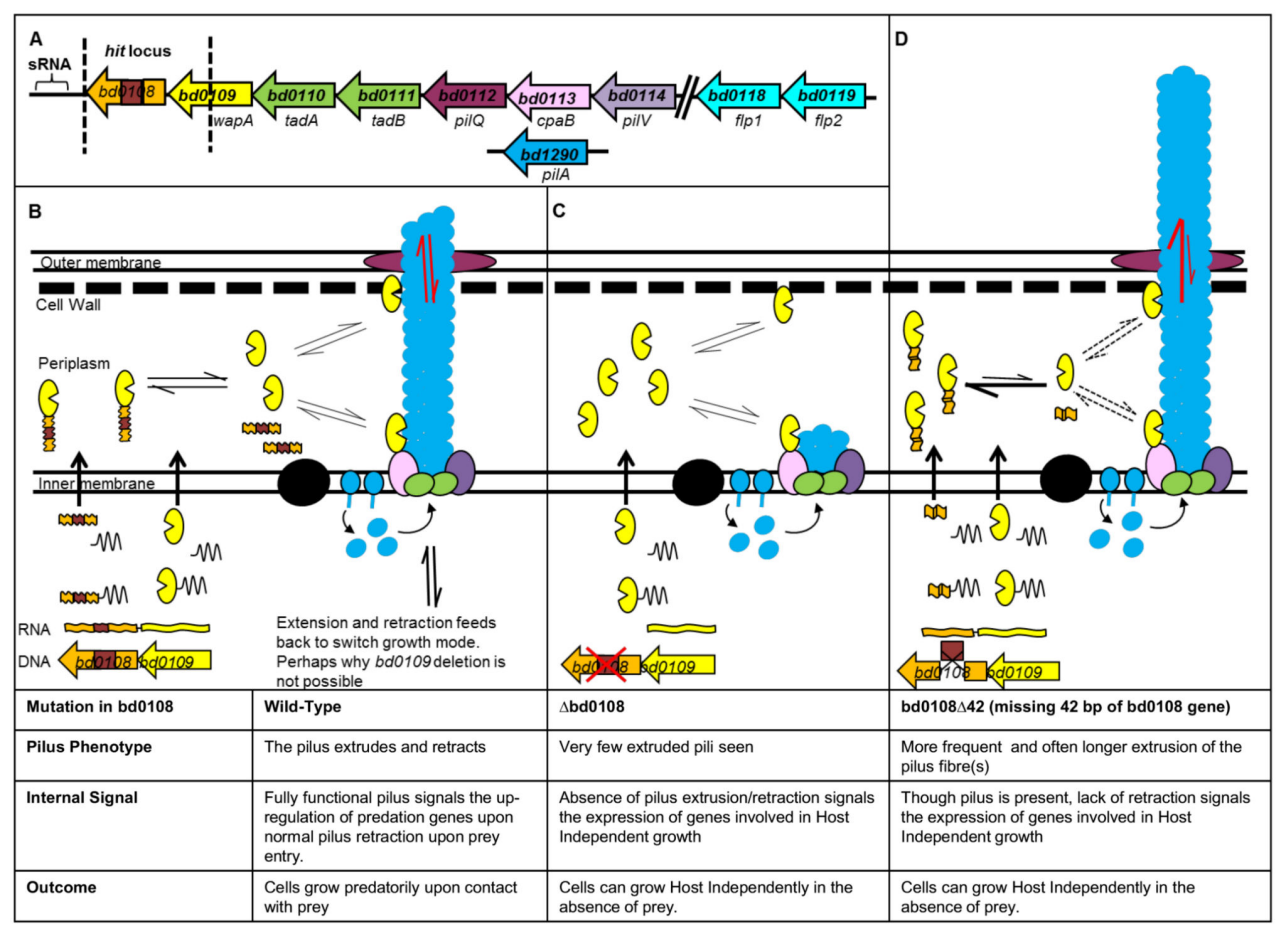
Model for possible interactions of Bd0108/Bd0109 controlling the extrusion and retraction of pili. A. Operonal structure of the *bd0108* *hit* locus and surrounding genes, predicted to have a role in the formation of a Type IVb pilus. Genes are colour coded to correspond to their predicted function in the pilus diagrams underneath. **B**. In wild-type cells *bd0108* and *bd0109* are co-expressed, the mRNA is then translated into proteins containing a signal sequence recognised by the Sec system, the signal is cleaved, and the proteins are transported into the periplasm where the mature Bd0108 protein transiently interacts with Bd0109 to sequester it. When Bd0109 is unbound, it could then anchor at either the cell wall, or with the mature pilus fibre. Both scenarios are possible due to Bd0109’s structural cleft binding carbohydrate that is present in both cell wall and the mature and glycosylated pili. Bd0109 mediates successful pilus extrusion/retraction and signal back into the cytoplasm. In wild-type pilus formation Bd1290 pre-pilins are held in the inner membrane and are assembled into the pilus fibre possibly by the flp pilus ATPases Bd0110 and Bd0111. The balance of sequestering and release of Bd0109 by Bd0108 in the periplasm permits to successful extrusion and retraction of the pilus fibre upon environmental cues. **C**. In the absence of Bd0108 protein, Bd0109 is not sequestered and is free to mediate more frequently with pilus extrusion and retraction, resulting in very few pili extruded beyond the cell surface and cues for HI growth signalled to the cell. **D**. In HI strains containing the 42 bp deletion variant of *bd0108*, the gene is still expressed. The truncated form of Bd0108 alters the dynamics of the Bd0109 functionalisation are altered (possibly by over-sequestration of Bd0109) and hyper-extruded pili are seen on the surface more frequently. Hyper-extruded pili or no pili send similar internal signals to regulate prey independent growth.

Thus we propose a model (Figure **11**) in which extrusion/retraction of a Type IVa pilus fibre sends signalling information into to the *Bdellovibrio* cell to cause it to grow and replicate. We postulate that this signal is sent when the *Bdellovibrio* invade prey (a Type IVa-pilus-dependent process); but also when growing as HI cultures in a (possibly pilus-associated) contact-dependent process that occurs at high cell densities and not in dilute cell suspensions. We propose that Bd0109 protein, modulated by Bd0108, regulates/monitors pilus extrusion/retraction post translationally. We propose that Bd0109 protein has an essential role by one of two means; 1) it is a recognition mechanism which transmits a growth signal by monitoring the extrusion/retraction of the pilus and without Bd0109, *Bdellovibrio* are non-viable as HI or HD cells because they don’t receive the growth signal. This signal could be an absence of normal extrusion/retraction that the attack phase *Bdellovibrio* would carry-out while seeking prey. This absence would be found in ∆*bd0108* mutants which have overly-retracted pili, the 42 bp deletion mutants of *bd0108* which have overly-extruded pili, and in wild-type attack phase cells having entered prey where pili would then be in a fixed state, possibly attached to the prey cell walls. The signal from Bd0109 could be transmitted into the *Bdellovibrio* cell by interactions with an as yet unidentified membrane protein. In this model, the Bd0109 is sequestered by Bd0108, and released in a stochastic manner to either interact at the cell wall with the extruding/retracting pilus, or in the presence of a static pilus, interact with an unknown membrane protein to signal cell growth. 

Alternatively 2) Bd0109 has a structural role, sited where pili and cell walls interact adjacent to PilQ and its absence destabilises the cell wall at this location, killing the cells. In this instance the pilus extrusion/retraction state is dependent on the Bd0109/Bd0108 interaction, but the growth signal is not. An unidentified signalling pathway would give the growth signal, responding to the level of pilus subunits inside the cell. 

Bd0109 has a carbohydrate binding motif and could thus bind to the peptidoglycan cell wall (possibly at a site the pilus has to pass through to be extruded) or to a sugar-modified form of the pilus fibre itself. It also has a predicted pilin-like fold which could interact directly with the forming pilus in a manner analogous to minor pilins causing extrusion or retraction [54]. Absence of some minor pilins, in other bacteria, has been shown to cause pili of aberrant lengths [60]. Alternatively, this pilin-like fold could sequester pilin chaperones causing stalling to alter the extrusion/retraction frequencies. Interaction of the Bd0108 protein with Bd0109 modifies this activity in a mechanism that would require the amino acids (73-RRHDDTVSREIKGSSATPGGSEKAGTGRQ-101), which are altered in the 42 bp deletion *bd0108* strain. This process could occur by the Bd0108 transiently sequestering Bd0109 in a stochastic and balanced manner to give the right amount of pilus extrusion and retraction in the wild-type upon environmental cues; a balance which is upset by the absence or truncation of Bd0108. The absence of Bd0108 could potentially lead to a larger pool of unsequestered Bd0109 which could then interact more frequently with the pilus causing more retraction and resulting in very infrequently seen extruded pili, which signals to the cell to enter growth phase. Truncation of Bd0108 such as by the 42 bp deletion form, commonly observed, could result in a stronger interaction between it and Bd0109, reducing the interaction between pili and Bd0109, leading to more and sometimes longer extruded pili, but resulting in the same signal releasing growth inhibition as with the Bd0108 deletion. The signal from correct pilus function, from either the binding or unbinding of a substrate could then pass into the cell to affect global transcription, possibly involving the small RNA that is differentially regulated according to *bd0108* genotype. 

Here, we have discovered in an unusual bacterium a novel regulation of pilus extrusion and retraction which sends a growth mode signal. This may have analogues in other bacteria and uncovers a novel role for the ubiquitous RHS family of proteins of which Bd0109 is a member. 

## Materials and Methods

### Growth of bacterial strains and generation of Host Independent isolates

*Bdellovibrio* and *E. coli* strains and plasmids in this study are listed in Table **5**. Predatory *Bdellovibrio* strains were maintained on Ca/HEPES buffer with *E. coli* S17-1 as prey as described previously [23,61,62]. All prey cells were grown in YT broth for 16 hours at 37°C with shaking at 200 rpm. Host-Independent (HI) strains were isolated as described previously [1,23] and maintained on PY agar and broth free of prey cells. Where appropriate, kanamycin sulphate (Apollo Scientific) was used at 50 µg ml^-1^ and IPTG (isopropyl-β-D-1-thiogalactopyranoside) was used for induction of fluorescent strains at 200 µg ml^-1^. 

**Table 5:** Bacterial Strains and Plasmids used in this study.

**Strain or Plasmid**	**Genotype or description**	**Reference**
***Escherichia coli***		
S17-1	*thi, pro, hsdR* ^-^, *hsdM* ^+^, recA; integrated plasmid RP4-Tc::Mu-Kn::Tn*7*; used as donor for conjugating plasmids into *Bdellovibrio*	[65]
BL21(DE3) pLysS	Strain for expressing genes under control of the T7 promoter	Promega corp.
***Bdellovibrio bacteriovorus***		
HD100	*Bdellovibrio* type strain, genome sequenced	[6,10]
HID2	HI isolate of HD100 with a wild-type *bd0108*	[27]
HID6	HI isolate of HD100 with a CC insertion after base 217 of *bd0108*	This study
HID13	HI isolate of HD100 with a G-A substitution at base 3 in *bd0108*	[27]
HID18	HI isolate of HD100 with a deletion of the base G at position 211 in *bd0108*	This study
HID22 (*bd0108∆42bp*)	HI isolate of HD100 with a 42 bp deletion from base 210 to base 252 in *bd0108*	[27]
HID26	HI isolate of HD100 with a wild-type *bd0108*	[27]
∆*bd0108*#1 ∆*bd0108*#2	HD100 with a markerless deletion of *bd0108*	This study
HD100 ∆*PilA*	HD100 ∆*pilA* with a markerless deletion of *bd1290*	[1]
**Plasmids**		
pZMR100	S17-1 strain containing pZMR100 plasmid used to confer Km^r^; used as Km^r^ prey for *Bdellovibrio*	[65,66]
pMAL-p2_mCherry	S17-1 containing an Amp^r^ plasmid containing a mCherry gene with a MalE signal sequence to localise fluorescence to the periplasm.	[47]
pCL100	Kn^r^ plasmid encoding the *luxR* operon for luminescence	[22]
pAKF56	pUC19 based Amp^r^ plasmid containing the *mCherry* for fluorescent tagging of genes	[47]
pAKF04	pUC19 based Kn^r^ plasmid containing the *mTFP* for fluorescent tagging of genes	[15]
pK18*mobsacB*	Kn^r^ suicide vector used for conjugation and recombination into the *Bdellovibrio* genome	[67]
pK18::108mCherry	pK18*mobsacB* vector containing C-terminally tagged mCherry fluorescent *bd0108*	This study
pK18::Δ42mCherry	pK18*mobsacB* vector containing C-terminally tagged mCherry fluorescent 42 bp deletion of *bd0108*	This study
pAKF04::109	pAKF04 vector containing C-terminally tagged mTFP fluorescent *bd0109*	This study
pK18::Δ108	pK18*mobsacB* a deleted *bd0108* and 1Kb 5’- and 3’- flanking DNA	This study
pK18::Δ109	pK18*mobsacB* a deleted *bd0109* and 1Kb 5’- and 3’- flanking DNA	This study
pZMR100	λ defective vector, Kn^r^. Used to confer Kn^r^ on S17-1 used as prey	[68]
pSUP404.2	Kn^r^ plasmids that replicates in *Bdellovibrio* and is used for complementation	[25]
pSUP404.2-108	pSUP404.2 containing *bd0108* and 200bp of 5’- and 3’- flanking	This study
pSUP404.2-Δ42	pSUP404.2 containing *bd0108* with the 42 bp deletion and 200bp of 5’- and 3’- flanking	This study
pK18::∆*bd0108*	pK18*mobsacB* suicide plasmid containing 1kb of 5’- and 3’- flanking genomic DNA from around *bd0108*	This study
pK18::∆*bd0109*	pK18*mobsacB* suicide plasmid containing 1kb of 5’- and 3’- flanking genomic DNA from around *bd0109*	This study
pET26b	Expression vector with *pelB* N-terminal fusion for export to periplasm and T7 promoter	Novagen

### Generating markerless deletion mutants

Markerless deletion of the *bd0108* open reading frame in *Bdellovibrio bacteriovorus* strain HD100 was achieved using a modified version of Steyert and Pineiro [63]:

Markerless gene deletion of the *bd0108* open reading frame was attained using a modified method of Steyert and Pineiro [63]. Approximately 1 KB of flanking DNA from the genes was amplified and fused together by PCR [with Phusion proof-reading polymerase (NEB)] using primers designed to leave only 2 codons from the start of the gene and 4 from the end, including the stop codon, after which a restriction site was incorporated (Table **6**). These PCR fusions were cloned into the vector pK18*mobsacB* in *E. coli* S17-1 and confirmed by restriction digest of plasmid preparations. The constructs were then conjugated into *Bdellovibrio bacteriovorus* HD100 as described elsewhere [22]. For Host Dependent strains, ex-conjugants were isolated by kanamycin (50 µg ml^-1^) selection on double layer overlay plates, with kanamycin resistant prey; *E. coli* S17-1 (pZMR100). To select for double crossovers, merodiploids were cultured in the absence of kanamycin and with 5% sucrose in predatory cultures for 24 hours. The *Bdellovibrio* were plaque purified on double layer overlay plates without kanamycin selection to screen for HD mutants. For HI isolates, ex-conjugants did not undergo sucrose selection but were filtered using a 0.45 µm filter to remove the remaining *E. coli*, and were concentrated by centrifugation and plated on PY agar plates to form HI colonies [1]. Resultant plaques or colonies were grown in the presence and absence of kanamycin to test for kanamycin sensitivity which confirmed a double crossover event (i.e. revertant to wild-type or generation of a deletion mutant). Any potential double crossovers were screened by Taq PCR (Bioline), and further confirmed by sequencing of a Phusion PCR product and by Southern blot hybridisation as well as RT-PCR analysis to confirm the absence of the gene transcript.

**Table 6:** Primers used in this study.

**Primer Name**	**Sequence 3’- 5’**	**Description**
**Cloning Primers**		
Bd0108-F	TTAAAACCATCACTGGCCCC	Forward Primer to amplify approximately 1 kb of upstream flanking DNA from *bd0108*
Bd0108-R	CTGTAGCATGCGATATGGTCATGCCTCTTCG	Reverse Primer to amplify approximately 1 kb of downstream flanking DNA from *bd0108*, with *Sph*I site added.
ΔBd0108-F	ATTATATGAAAGGAAGACAGTAAGGATCCTCCCATCTG	Internal primer with homology to the 5’ start and 3’ end of the gene *bd0108*, with *Bam*HI site added
ΔBd0108-R	ATGCGGGATCCTTACTGTCTTCCTTTCATATAATCACCTTCTC	Internal primer with homology to the 5’ start and 3’ end of the gene *bd0108*, with *Bam*HI site added
Bd0109F	CGATGGAATTCGCAAAACCACCAATCCCAGC	Forward Primer to amplify approximately 1 kb of upstream flanking DNA from *bd0109*, with *Eco*RI site added.
Bd0109R	CGATGCATGCGGTGTCTTAGCGAATCGCCT	Reverse Primer to amplify approximately 1 kb of downstream flanking DNA from *bd0109*, with *Sph*I site added.
Bd0109ΔF	TCAAGGAAGAGGTATCGATAGGTACCGGAGAAGGTGATTATATG	Internal primer with homology to the 5’ start and 3’ end of the gene *bd0109*, with *Kpn*I site added
Bd0109ΔR	CATATAATCACCTTCTCCGGTACCTATCGATACCTCTTCCTTGA	Reverse Primer to amplify approximately 1 kb of downstream flanking DNA from *bd0109*, with *Kpn*I site added.
Bd0108-Fcomp	GGATTCCTAAAATCGAGTACGAGGAG	Forward primer to amplify approximately 200 bp of upstream flanking of DNA from *bd0108*
Bd0108-Rcomp	GGAATTCCTAGCAATAGTGGCTTGTACG	Reverse primer to amplify approximately 200 bp of upstream flanking of DNA from *bd0108*
Bd0108mCherryR	GGGGTACCCTGTCTTCCAGTCCCGGCTT	Reverse primer used to amplify *bd0108* for cloning into pAKF56 and subsequent gene fusion with the mCherry gene
Bd0109mtfpR	TACGTAGGGGTACCCAGGTAAAGTTCCGCAGTTG	Reverse primer used to amplify *bd0109* for cloning into pAKF04 and subsequent gene fusion with the mTFP gene
Bd0108_SP_Fwd	CCTCGCTGCCCAGCCGGCGATGGCCGACGAAAATGCCAACCGCCCGGTAAACC	Forward primer for restriction free cloning of *bd0108* into expression vector
Bd0108_SP_Rev	CTCAGTGGTGGTGGTGGTGGTGCTCGAGCTGTCTTCCAGTCCCGGCTTTCTC	Reverse primer for restriction free cloning of *bd0108* into expression vector
Bd0109_SP_Fwd	CCTCGCTGCCCAGCCGGCGATGGCCCTTGTGGATATGAAAAATGCCAACTACTCC	Forward primer for restriction free cloning of *bd0109* into expression vector
Bd0109_SP_Rev	CTCAGTGGTGGTGGTGGTGGTGCTCGAGCAGGTAAAGTTCCGCAGTTGCGGG	Reverse primer for restriction free cloning of *bd0108* into expression vector
**RT-PCR Primers**		
Bd0108-42RTF	AAAGACTCTTGGTCCTTTCC	Forward internal primer used to detect transcription of *bd0108* by RT-PCR. The primer flanks the region of *bd0108* where the 42 bp deletion arises.
Bd0108-42RTR	GCTTTCTCAGATCCACCAGG	Reverse internal primer used to detect transcription of *bd0108* by RT-PCR. The primer flanks the region of *bd0108* where the 42 bp deletion arises.
dnaK-RTF	TGAGGACGAGATCAAACGTG	Forward internal primer used to detect transcription of *dnaK* by RT-PCR.
dnaK-RTR	AAACCAGGTTGTCGAGGTTG	Forward internal primer used to detect transcription of *dnaK* by RT-PCR.
Bd0109-RTF	ACTATCCTGATGGCCTGGTG	Forward internal primer used to detect transcription of *bd0109* by RT-PCR.
Bd0109-RTR	GATGCTGTTCACCGAACTGA	Reverse internal primer used to detect transcription of *bd0109* by RT-PCR.
mCherry-R	GAGCCGTACATGAACTGAGG	Reverse internal primer used to detect transcription of mCherry gene fusions by RT-PCR.
mTFP-R	CTCCAGGTTGATGGTGTTGG	Reverse internal primer used to detect transcription of mTFP gene fusions by RT-PCR.
103-108sRNAF	CTCTTGTTTGAACGCTGTCG	Forward internal primer used to detect transcription of small RNA between *bd0103* and *bd0108* by RT-PCR.
103-108sRNAR	TGTCTTTGATCGCCTCTGTG	Reverse internal primer used to detect transcription of small RNA between *bd0103* and *bd0108* by RT-PCR.
Bd0110F	CCATGAAACGTGTTGTGGAG	Forward internal primer used to detect transcription of *bd0110* by RT-PCR.
Bd0110R	TCAAAGCTGTCACCCACTTG	Reverse internal primer used to detect transcription of *bd0110* by RT-PCR.
TadA RT-F	GACAAGGGTGTTGTGATTCC	Forward internal primer used to detect transcription of *bd0111* by RT-PCR.
TadA RT-R	ATTTTCGGCGCCATCGCAGC	Reverse internal primer used to detect transcription of *bd0111* by RT-PCR.

### Complementation of markerless gene deletion

Complementation of gene deletions in *Bdellovibrio* was possible using the replicating plasmid pSUP404.2 containing the wild-type gene for that knock-out strain, with 200 bp of flanking DNA allowing for the inclusion of promoters and terminators [25]. Using this method it was possible to clone *bd0108* and *bd0108* with the 42 bp deletion into the *Eco*RI site of pSUP404.2. These plasmids, and empty vector, were transformed into *E. coli* S17-1 and conjugated into the *Bdellovibrio* strains: HD100, HID13, HID22, *Δbd0108*#1, and *Δbd0108*#2, with all exconjugants being grown in predatory culture in the presence of *E. coli* S17-1 (pZMR100) as prey under kanamycin selection.

### Fluorescent tagging of Bd0108 and Bd0109

Using the fluorescent plasmid pAKF56 [47] containing a *mCherry* gene tag and pAKF04 [15] containing an *mTFP* gene tag it was possible to fluorescently tag Bd0108 (mCherry), Bd0108-Δ42 (mCherry) and Bd0109 (mTFP). The genes were cloned into the respective plasmids without the stop codons with direct fusion of the gene to the fluorescent tag genes at the C-terminus. Amplification of the *bd0108* gene, its *bd0108* 42 bp deletion variant or *bd0109* was carried out with Phusion polymerase with primers incorporating a restriction site at the start and end of the of the gene, the stop codon was missed out to allow read through into the fluorescent tag (Table **6**). The amplified gene products were cloned into pAKF56 (*bd0108*, *bd0108* 42 bp deletion) or pAKF04 (*bd0109*) in *E. coli* S17-1, as described previously [15,47]. 

The fluorescent plasmids were designed to integrate into the genome by single crossover events to utilise the natural promoter of the target gene.

Correct insertion was verified by fluorescence microscopy, restriction digests and sequencing of the plasmid. The genes were excised with the fluorescent tag and cloned into pK18*mobsacB*, transformed into S17-1, and then conjugated into wild-type *Bdellovibrio bacteriovorus* HD100 as well as the *Δbd0108* markerless deletion mutant, with ex-conjugants being selected for by kanamycin sulphate (50 µg ml^-1^). Kanamycin selection was kept to maintain integration of the tagged gene in the *Bdellovibrio* genome; the area of integration was sequenced and subsequent fluorescent analysis was then performed. 

### Plaquing assay

*Bdellovibrio* strains; HD100, HID13, HID22, Δ*bd0108* #1 and #2, containing the plasmid pSUP404.2, pSUP404.2-108, or pSUP404.2-Δ42 were grown under kanamycin selection (50 µg ml^-1^) in predatory cultures utilising S17-1 (pCL100) as a prey [22]. Once the cultures were fully lysed 100 µl of each strain was taken for serial dilution, 10^0^-10^-4^ in Ca/HEPES buffer. Ten microlitres of each serial dilution was then taken and spotted on pre-poured double layer YPSC plates already containing prey in the top layer of agar. Each dilution was spotted equidistant from one another and allowed to air dry in a Class II hood. Plates were then incubated (agar on the bottom) statically at 29°C for 7 days.

### RNA isolation from predatory cycle and RT-PCR analysis

Synchronous predatory infections of *Bdellovibrio bacteriovorus* HD100 on *E. coli* S17-1 as well as a S17-1 alone control were set up as previously described [23] with samples throughout the timecourse being taken and total RNA isolated from them. For HI strains, growing free of prey cells, the samples were taken after back dilution and subsequent growth to OD_600_ of 0.6 as described previously [1].

RNA was isolated from the samples using a Promega SV total RNA isolation kit with the RNA quality being verified by an Agilent Bioanalyser using the RNA Nano kit. RT-PCR was performed with the Qiagen One-step RT-PCR kit with the following reaction conditions: One cycle 50°C for 30 mins, 95°C for 15 mins, then 25 cycles of 94°C for 1 min, 48°C for 1 min, 72°C for 2 mins, and finally a 10 mins extension at 72°C after the 30 cycles, and finally a 4°C hold. All primers are listed in Table **6**. All experiments were carried out with at least 2 biological repeats.

### RNA-seq analysis

RNA was isolated as described above and 10 µg of each sample was used before rRNA depletion for library construction. Transcriptome libraries were constructed using the Illumina TruSeq RNA sample preparation kit with modifications. The rRNA was depleted using Epicentres Ribo-Zero™ rRNA Removal Kit for Gram-Negative Bacteria. The rRNA removal was confirmed with a Pico chip run on the Agilent Bioanalyser 2100 and the quantity measured with the Qubit RNA kit and Qubit fluorometer (Invitrogen). The resulting ribosomal depleted RNA was then fragmented for 8 minutes at 94°C using the Elute, Fragment, Prime buffer from Illumina TruSeq RNA kit. These conditions give final libraries of around 400bp. The samples were then processed following the standard TruSeq RNA protocol. Each library pool was diluted to 2 nM with NaOH and 5μL transferred into 995 µl HT1 to give a final concentration of 10 pM. One hundred and twenty microlitres of normalised library was then transferred into a 200 μL strip tube and placed on ice before loading onto the Illumina cBot. Flow cells were clustered using TruSeq Single-Read Cluster Generation Kit V2, using the SR_Amp_Lin_Block_Hyb_v8.0 recipe. Following the clustering procedure, the flow cell was loaded onto the Illumina HiSeq2000 instrument following the manufacturer’s instructions. The sequencing chemistry used was TruSeq SBS version3 using Illumina software HCS 1.4.8 and RTA 1.12.4.2.

Reads were mapped to the *Bdellovibrio bacteriovorus* HD100^T^ genome (Genbank accession number NC_005363.1) and transcripts were quantified and analysed for differential expression as described previously [64]. The data has been submitted to the European Nucleotide Archive with the accession number ERP001980.

### Electron microscopy

HI *Bdellovibrio* were grown overnight to an OD_600_ of 0.1-0.5 in PY broth at 29°C with shaking for 18-24 hours. Predatory *Bdellovibrio* strains were maintained on Ca/HEPES buffer with *E. coli* S17-1 as prey as described previously [23,61]. Cells with the pSUP404.2 plasmid or derivatives were maintained on *E. coli* S17-1 (pZMR100) as prey with 50 µg ml^-1^ kanamycin sulphate. Two Microlitre samples were stained with 10 µl 2% phosphotungstic acid (PTA) solution pH 7.0 for 1 minute on Veco copper grids, 200 mesh. Cells were observed with a JEOL 1200Ex electron microscope at 80 kV.

### Predation assays

The Host Dependent HD100 and the HI strains were grown in predatory cultures using *E. coli* S17-1 as prey and PY broth respectively and were matched by Lowry protein assay [16] to 500 µg total, which was then used to prey upon S17-1 matched to OD_600_ 1.0 in Ca/HEPES and predation monitored over 48 hours, with enumerations of the S17-1 being taken every 24 hours.

Luminescent predation assays for wild-type *Bdellovibrio* HD100 and the spontaneously isolated and markerless deletion of *bd0108*, as well as complementation with pSUP404.2 containing the genes; *bd0108*, and *bd0108* with the 42 bp deletion, were carried as previously stated [22]. All strains were grown in predatory culture with *E. coli* S17-1 (pCL100) as prey. Enumerations of each *Bdellovibrio* strain were carried out by PFU on double-layer overlay plates and as CFU on PY agar plates. The assay was carried out using a BMG labtech Fluostar plate reader at 29°C for 48 hours with shaking at 200 rpm and luminescence readings being taken every 15 minutes. 

### Fluorescent/time-lapse microscopy

Epi-fluorescence microscopy was undertaken using a Nikon Eclipse E600 through a 100x objective (NA 1.25) and acquired using a Hammamatsu Orca ER Camera. The microscope was also fitted with a Prior Scientific H101A XYZ stage for revisiting the same field of view over time-lapse experiments. Images were captured using Simple PCI software (version 5.3.1).Time-lapse microscopy was carried out as described previously [47]: Predatory liquid cultures of *Bdellovibrio bacteriovorus* HD100 (pSUP404.2) and Δ*bd0108* (pSUP404.2) were grown in 10 ml Ca/HEPES, with kanamycin selection (50 µg ml^-1^) in with *E. coli* S17-1 (pMAL-p2_mCherry), synchronous predatory cultures were then set up after complete lysis of prey cells, as described previously [47]. One millilitre of the predatory culture was centrifuged and resuspended in 100 µl Ca/HEPES; 1 ml of an overnight culture of *E. coli* S17-1 (pMAL-p2_mCherry) (Kanamycin 50 µg ml^-1^, IPTG 200 µg ml^-1^) was centrifuged and resuspended in 100 µl Ca/HEPES. The *Bdellovibrio* and the *E. coli* were mixed, and 10 µl of the cell mixture was spotted onto a 1% agarose/Ca-HEPES (w/v) pad on a microscope slide, to immobilise the cells, and coverslip placed on top. Time-lapse fluorescent microscopy was undertaken using a Nikon Eclipse E600 with a 100x objective lens and a mounted Hammamatsu Orca ER Camera as well as an hcRED filter block (excitation: 550 to 600 nm; emission: 610 to 665 nm); the microscope was also equipped with a Prior Scientific H101A XYZ stage to allow the camera to revisit previous fields of view every 150 seconds (2.5 minutes). All images and analysis were captured using Simple PCI software (version 5.3.1).

For *E. coli* fluorescence of the tagged genes *bd0108* and *bd0109*, *E. coli* S17-1 containing the plasmids pK18::108mCherry, pK18::Δ42mCherry and pAKF04::109, were grown for 16 hours in YT broth containing 50 µg ml^-1^ kanamycin (pK18::108mCherry, pK18::Δ42mCherry) or 50 µg ml^-1^ ampicillin (pAKF04::109). Ten microlitres of each was spotted onto a 1% agarose/Ca-HEPES (w/v) pad on a microscope slide and a coverslip placed on top. Images were taken using the equipment as described above, with only single stills being taken instead of time-lapse. For those strains containing pAKF04::109 a CFP filter was used (excitation: 458-500nm; emission 420-454). 

For localisation of fluorescently tagged proteins an hcRED filter block (excitation: 550-600 nm; emission: 610-665 nm), for mCherry tags or a CFP filter (excitation: 458-500nm; emission 420-454), for mTFP tags was used.

### Shearing and SDS-PAGE analysis of pilus composition

HI strains were grown host dependently on *E. coli* S17-1 prey cells in Ca/HEPES buffer before being concentrated 100x by centrifugation at 4233 x g for 30 minutes and resuspended in 2.5 ml of Ca/HEPES buffer. The cells were passed through a 30 cm long size 3 FG cannulum (Portex) twenty times to shear off extracellular organelles [43]. Cells were removed from the sample by centrifugation at 17,000 x g for 2 minutes and filtration through 0.2 µm filters. Supernatants containing extracellular organelles were subjected to centrifugation at 88,000 x g for 2 hours and pellets were resuspended in 50 µl TE. 30 µl samples were added to 10 µl 4x Tris loading buffer and incubated at 37°C for 30 minutes [44] before being loaded onto a Tris-Tricene precast gel (10-20%; BioRad) and ran at 30 V for 30 minutes until the samples had entered the resolving gel, then increased to 50-90 V until the sample dye reached the end of the gel. The gels were stained with Coomassie Brilliant Blue Stain and destained with 30% methanol/10% acetic acid solution. Bands were cut out of the gel and sent for LC-MS/MS analysis.

### Expression and purification of Bd0108 and Bd0109

Constructs expressing signal peptide exported variants of Bd0108 and Bd0109 were prepared using a restriction-free cloning protocol, placing residues D24-Q101 of Bd0108 and L19-L550 of Bd0109 into the pET26b vector; the coding sequence inserted such that the proteins immediately followed the signal peptidase cleavage site and contained a C-terminal LEHHHHHH-ter affinity tag. Transformed cells were grown in 1 litre of 2xYT media (shaking at 37°C) until they reached an approximate OD_600_ of 0.7 upon which protein expression was induced using 0.4 mM IPTG (temperature shift to 21°C, left shaking overnight). Pelleted cells were resuspended in 45 ml of buffer A (20 mM HEPES pH 7.2, 300 mM NaCl, 0.1% w/v Tween-20, 20 mM imidazole, 8% w/v glycerol), lysed via six 20 second cycles of sonication and centrifuged for 60 minutes at 200,000x g. The supernatant was applied to a 1 ml Hi-Trap nickel affinity column (Amersham Biosciences), and peak fractions eluted using a step gradient of buffer A supplemented with 50 and 300 mM imidazole. Fractions containing the desired protein were dialysed into 30 mM Tris pH 8.0, 300 mM NaCl and 8% w/v glycerol.

### Fluorescence quenching Measurement

We performed a fluorescence emission (F_emission_) scan of Bd0109 in solution by recording (F_emission_) output between 300 and 400 nm, upon excitation at lambda_285nm_. Bd0109 gave a maximum fluorescence emission (F_emission_^max^) at a wavelength of 340 nm, thus providing a basal F_emission_ coordinate for the collection of subsequent Intrinsic Tryptophan fluorescence (ITF) data. The change in fluorescence emission (DF_emission_) was calculated by subtracting the F_emission_ (recorded 5 minutes after each ligand titration) away from (F_emission_^max^), and the data was then plotted against [Bd0108]. Nonlinear regression analysis of DF_emission_ vs [Bd0108], using the following equation: D*F*_*emission*_ = *F*_*max*_ x *L* / (*K*_*d*_ + *L*), shows a hyperbolic isothermal ligand binding curve for Bd0108. 

## Supporting Information

Figure S1 **Mutations in *bd0108* of various *Bdellovibrio bacteriovorus* isolates and the effect on the resultant amino acid sequence.** Wild-type amino acid sequence is shown in blue, while additional or altered amino acids are in red. HID2 and HID26 both have wild-type copies of *bd0108* in their genome. In the case of HID6 and HID18 insertion and deletion respectively alter the amino acid sequence from wild-type and also results in an altered stop codon further downstream. Though HID22 (*bd0108∆*42bp) has undergone a 42 bp deletion (removing -RRHDDTVSREIKGS-) the reading frame is still maintained. In the case of ∆*bd0108* all that remains of the Bd0108 protein sequence is the first two amino acids and the last three together with the original stop codon.
(TIF)

Figure S2 **Tricine-PAGE of sheared protein preparations of Host Independent strains grown in predatory cultures.** Indicated bands are more pronounced in the HID22 (*bd0108∆*42bp). When subjected to Mass Spectrophotometry the band indicated by the red arrows at ~20 kDa had products with homology to various *Bdellovibrio* and *E. coli* proteins but importantly to PilA (Bd1290) coinciding with the presence of large pilus structures seen in electron micrographs (**Figure 9**.) and suggesting that these structures are likely made of PilA. This band was not detectable in the Δ*bd0108* HI isolates corresponding with the almost totally absence of pili seen in this strain by electron micrographs. The black arrowed band at 17 kDa also had products with homology to a variety of different *Bdellovibrio* and *E. coli* proteins including flagellin and OmpA.
(TIF)
