# Supplementary figures and images for: Activity of Bdellovibrio Hit Locus Proteins, Bd0108 and Bd0109, Links Type IVa Pilus Extrusion/Retraction Status to Prey-Independent Growth Signalling
Source: PLoS One. 2013 Nov 5;8(11):e79759. doi: 10.1371/journal.pone.0079759 (PMC3818213; doi:10.1371/journal.pone.0079759)

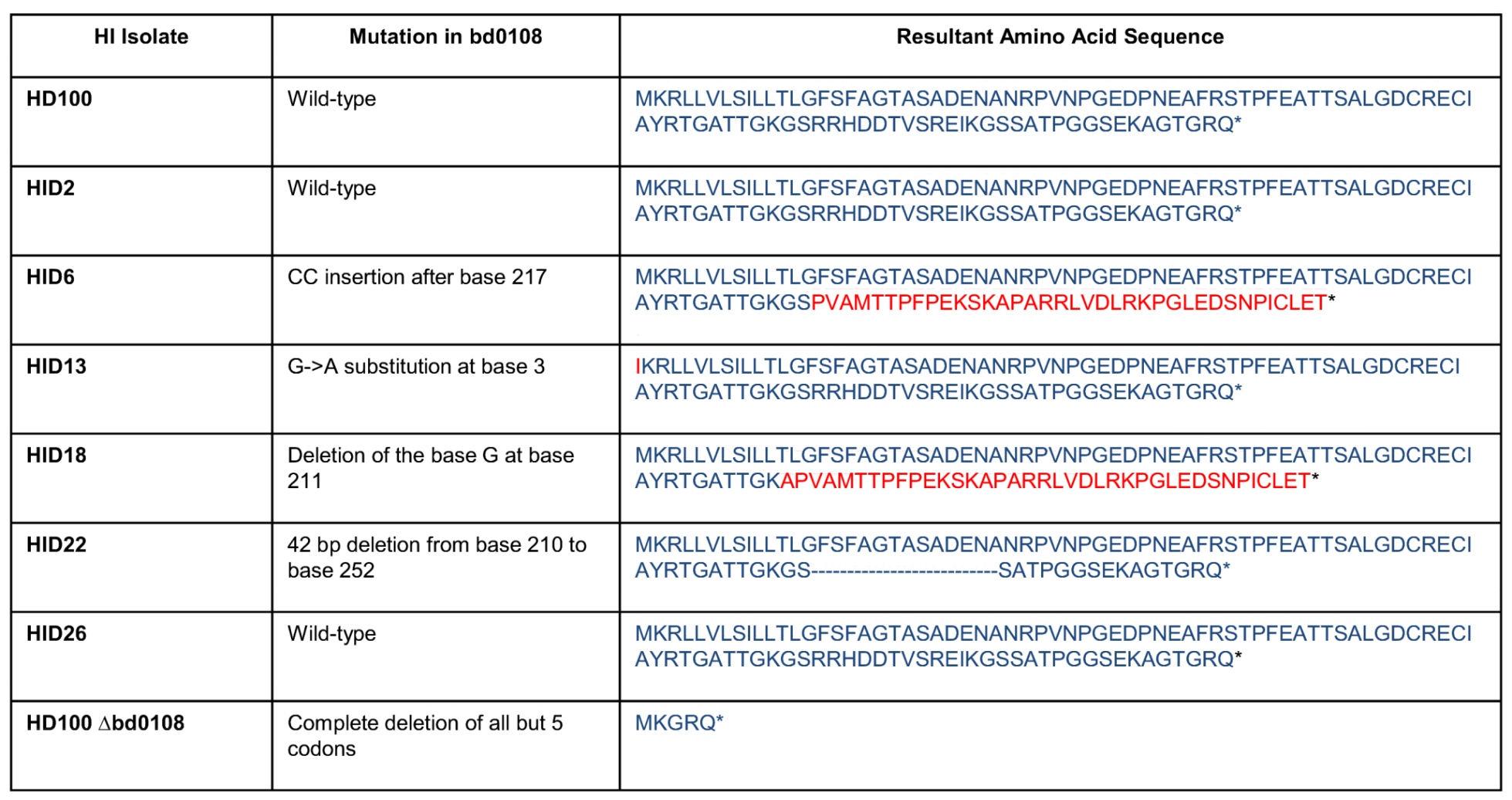

Supplement: Figure S1 — Mutations in bd0108 of various Bdellovibrio bacteriovorus isolates and the effect on the resultant amino acid sequence. Wild-type amino acid sequence is shown in blue, while additional or altered amino acids are in red. HID2 and HID26 both have wild-type copies of bd0108 in their genome. In the case of HID6 and HID18 insertion and deletion respectively alter the amino acid sequence from wild-type and also results in an altered stop codon further downstream. Though HID22 (bd0108∆42bp) has undergone a 42 bp deletion (removing -RRHDDTVSREIKGS-) the reading frame is still maintained. In the case of ∆bd0108 all that remains of the Bd0108 protein sequence is the first two amino acids and the last three together with the original stop codon. (TIF) [file pone.0079759.s001.tif]

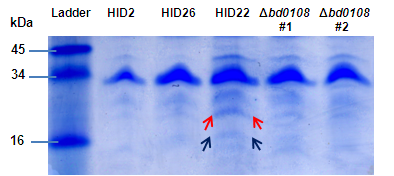

Supplement: Figure S2 — Tricine-PAGE of sheared protein preparations of Host Independent strains grown in predatory cultures. Indicated bands are more pronounced in the HID22 (bd0108∆42bp). When subjected to Mass Spectrophotometry the band indicated by the red arrows at ~20 kDa had products with homology to various Bdellovibrio and E. coli proteins but importantly to PilA (Bd1290) coinciding with the presence of large pilus structures seen in electron micrographs (Figure 9.) and suggesting that these structures are likely made of PilA. This band was not detectable in the Δbd0108 HI isolates corresponding with the almost totally absence of pili seen in this strain by electron micrographs. The black arrowed band at 17 kDa also had products with homology to a variety of different Bdellovibrio and E. coli proteins including flagellin and OmpA. (TIF) [file pone.0079759.s002.tif]
